# YOLO-RSTS: a precise segmentation model for detecting preservative and stimulant spraying regions on rubber trees

**DOI:** 10.3389/fpls.2025.1738496

**Published:** 2026-01-07

**Authors:** Jincan Zhu, Yu Feng, Fengming Liu, Lee Seng Hua, Haocen Zhao, Bangqian Chen, Weili Kou, Jian Rong, Guiliang Chen, Dingfei Xu

**Affiliations:** 1 College of Big Data and Intelligent Engineering, Southwest Forestry University, Kunming, Yunnan, China; 2 Yunnan International Joint Laboratory of Natural Rubber, Kunming, Yunnan, China; 3 Key Laboratory of Forestry Ecological Big Data of State Forestry and Grassland Administration, Southwest Forestry University, Kunming, Yunnan, China; 4 Department of Wood Industry, Faculty of Applied Sciences, Universiti Teknologi MARA (UiTM) Pahang Branch, Bandar Tun Razak, Pahang, Malaysia; 5 Institute for Infrastructure Engineering and Sustainable Management (IIESM), Universiti Teknologi Majlis Amanah Rakyat (MARA) (UiTM), Shah Alam, Selangor, Malaysia; 6 Rubber Research Institute, Chinese Academy of Tropical Agricultural Sciences, Haikou, Hainan, China; 7 Yunnan Institute of Tropical Crops, Chinese Academy of Tropical Agricultural Sciences, Jinghong, Yunnan, China; 8 Yunxiang Investment Co., Ltd., Luang Namtha, Lao People's Democratic Republic

**Keywords:** automated latex plantation management, CG-Attention mechanism, CrossScaleDSC and C2f-DSC module, spray of preservatives and ethylene, YOLO-RSTS

## Abstract

The application of preservatives and ethylene stimulants is critical for improving latex yield and extending the lifespan of rubber trees; however, traditional manual spraying methods are inefficient and unsuitable for large-scale plantation management. Moreover, existing segmentation models are challenged by complex bark textures and varying illumination conditions, resulting in blurred spraying boundaries and reduced recognition accuracy. To address these issues, this study proposes an improved segmentation model based on the YOLOv12n-Seg framework, termed YOLO-RSTS (YOLO for Rubber Spraying Target Segmentation), for accurately distinguishing preservative and stimulant spraying regions on rubber trees. The proposed model introduces three novel modules: CrossScaleDSC, CG-Attention, and C2f-DSC, which enhance long-range dependency modeling, suppress background noise through combined spatial–channel attention, and enable fine-grained multi-scale feature extraction with low computational complexity. In addition, RFCAConv and DWConv are incorporated into the backbone and head to strengthen spatial diversity and contextual representation. Experiments conducted on a self-constructed dataset demonstrate that YOLO-RSTS significantly outperforms the baseline YOLOv12n, achieving improvements of 6.3% in Precision (from 0.819 to 0.882), 6.3% in mAP0.50 (from 0.788 to 0.851), and 8.1% in Recall (from 0.740 to 0.821), while reducing the parameter count by 14.5% (from 2.72M to 2.33M). Meanwhile, compared with the latest YOLOv13n, YOLO-RSTS also achieves superior performance, with increases of 7.5% in mAP0.50 and 9.2% in F1 score. These results indicate that the proposed method provides an effective and efficient solution for vision-based autonomous spraying and holds significant potential for advancing intelligent rubber plantation management.

## 1 Introduction

Derived from Hevea brasiliensis, natural rubber is a versatile biopolymer integral to vehicle manufacture, medical instrumentation, industrial components and everyday consumer products, rendering it a critical strategic resource for industrial economies worldwide (Nair, 2021). The rubber industry not only generates substantial economic returns for major exporting countries but also plays an important role in supporting rural employment and sustainable development. Data indicate that global demand for natural rubber rose modestly in 2023 to about 14.82 million tons, representing roughly a 1.4% year-on-year increase. IMARC estimates the global natural rubber market at approximately USD 19.5 billion in 2024 and forecasts a compound annual growth rate (CAGR) of about 4.72% from 2025 to 2033, underscoring the sector’s solid long-term growth prospects (IMARC Group, 2024). Moreover, Brazil’s rubber plantations have expanded beyond 10 million hectares at present, and this rapid scale-up has positioned Brazilian-grown rubber as a dominant source in the global market, accounting for roughly 40% of world natural rubber supply (Thomas, 2016). This trend of continued expansion and development in global rubber production has heightened the industry’s prominence in the economies and ecosystems of tropical developing countries, while also posing unprecedented challenges for the automated management of large-scale rubber plantations.

Against this backdrop, the latex yield and health of rubber trees have become core issues in cultivation management. To increase latex yield, growers typically apply ethylene-releasing agents (such as Ethephon) before tapping to promote latex secretion. This method can significantly extend the secretion cycle of the tapping cuts, usually maintaining latex secretion in about 60% of the tapped areas (Kumari and Nugawela, 2013; Florez-Velasco et al., 2024). However, relying solely on the use of growth regulators is not enough to ensure the long-term health and efficient latex production of rubber trees. During tapping, if the cuts are not promptly treated, they are highly susceptible to fungal and bacterial infections, leading to bark rot, which in turn reduces latex yield and even shortens the lifespan of the rubber tree (Liu et al., 2019). Therefore, in addition to the application of growth regulators, it is also necessary to promptly spray antifungal agents on the cuts after tapping. These two steps—promoting latex secretion and applying antifungal agents—are indispensable key measures in ensuring the health of rubber trees and achieving sustained high yield (Upadhyay et al., 2024).

With the continuous advancement of agricultural automation and artificial intelligence technologies, new solutions have emerged for rubber plantation management. Precision spraying systems based on machine vision can direct spray application to target areas through image recognition, thereby improving efficiency and optimizing resource utilization. For example, the intelligent recognition system based on YOLOv4, developed by Arjun Upadhyay and others, achieved a 93.33% effective spraying rate and 100% recognition accuracy in indoor experiments (Hussain et al., 2020); The YOLOv3-tiny variable-rate spraying system proposed by Hussain and others saved up to 42% of pesticide usage (Wang et al., 2024). Yong Wang and his team combined the leaf area index (LAI) with wind speed control mechanisms, achieving a spraying response time of only 2.5 seconds in the field, with a control accuracy exceeding 95.9% (Chavan, 2019). Paolo Sanchez and his team deployed a lightweight SSD-MobileNetV1 model on the Jetson Nano platform, enabling efficient image recognition and real-time spraying control on embedded devices (Sanchez and Zhang, 2022).

Moreover, drone (UAV) spraying technology has achieved significant results in field crops and fruit tree pest management in recent years, effectively reducing the impact of pesticide application on human health (Khan et al., 2021; Sassu et al., 2024; Gatkal et al., 2025; Sahni and Mikeska, 2025). However, due to the dense canopy and narrow operating space of rubber plantations, its application still faces limitations. In contrast, ground-based mobile spraying robots, with their higher path flexibility and positioning accuracy, are more adaptable to the complex environment of rubber plantations. For example, Ashish T. Meshram and others develop4090ed a ground-based directional spraying robot based on convolutional neural networks (CNN), capable of automatically identifying plant pests and spraying as needed, showing good environmental friendliness and operational efficiency (Meshram et al., 2022). Madeleine Darbyshire and others proposed the practical application of object detection algorithms for weed spraying in precision agriculture, exploring the comparison of different algorithms in precision spraying (Darbyshire et al., 2023); Adrian Salazar-Gomez, on the other hand, evaluated the trade-offs between accuracy and real-time performance of weed detection systems through cross-dataset and platform comparisons, providing optimization ideas for agricultural automation spraying systems (Salazar-Gomez et al., 2022).

Although significant progress has been made in the identification of field crops, orchards, and weeds, two key issues remain: First, many datasets used for other crops are generally small in scale and limited in data volume, which means that despite high recognition accuracy, the generalization ability and practical application effectiveness of these models remain constrained. Second, research on intelligent spraying systems for the specific environment of rubber plantations, particularly for the management of antifungal spraying in tapping areas, is still in its relatively insufficient stages. Currently, existing technologies generally lack the ability to accurately distinguish and identify the antifungal and ethylene induction spraying areas on rubber trees, resulting in limitations in the accuracy and efficiency of spraying operations. Therefore, enhancing the precise localization capability of the rubber spraying system, the timeliness of automatic recognition, and achieving accurate differentiation between antiseptic and oxytocic areas have become urgent technical challenges that need to be addressed.

To address the existing challenges, this study plans to introduce a specialized dataset specifically focused on rubber tree spraying scenarios and develop an advanced segmentation model, YOLO-RSTS. Building on the YOLOv12n framework, the model will incorporate three custom-designed modules—CrossScaleDSC, CG-Attention, and C2f-DSC—to enhance global context awareness and long-range feature dependencies. These innovations will improve the separation of spatial and channel features while optimizing multi-scale feature extraction, enabling precise segmentation of both antiseptic and oxytocic areas on rubber trees. Additionally, the RFCAConv and DWConv modules will be integrated into the backbone and neck to boost robustness under varying lighting conditions and complex bark textures. These planned improvements aim to significantly enhance segmentation accuracy, offering strong support for intelligent latex plantation management. The key contributions of this research include:

1. A novel YOLO-RSTS model is proposed, specifically designed for high-precision segmentation of preservative and stimulant spraying target regions in rubber plantation management.
2. Three self-designed modules CrossScaleDSC, CG-Attention, and C2f-DSC are integrated to improve global context awareness, multi-scale feature extraction, and noise suppression.
3. RFCAConv and DWConv modules are further incorporated into the backbone and neck networks, improving spatial diversity and contextual feature representation.
4. Extensive experiments on a self-constructed spraying segmentation dataset demonstrate that the proposed model achieves a 6.3% improvement in accuracy, 6.3% increase in mAP0.50, and 8.1% gain in recall, while reducing the number of parameters by 14.5% compared to YOLOv12n-Seg.
5. This work offers a robust visual solution for intelligent spraying systems, advancing the automation and smart management of latex plantation practices.

## 2 Methods

**Figure 1:**
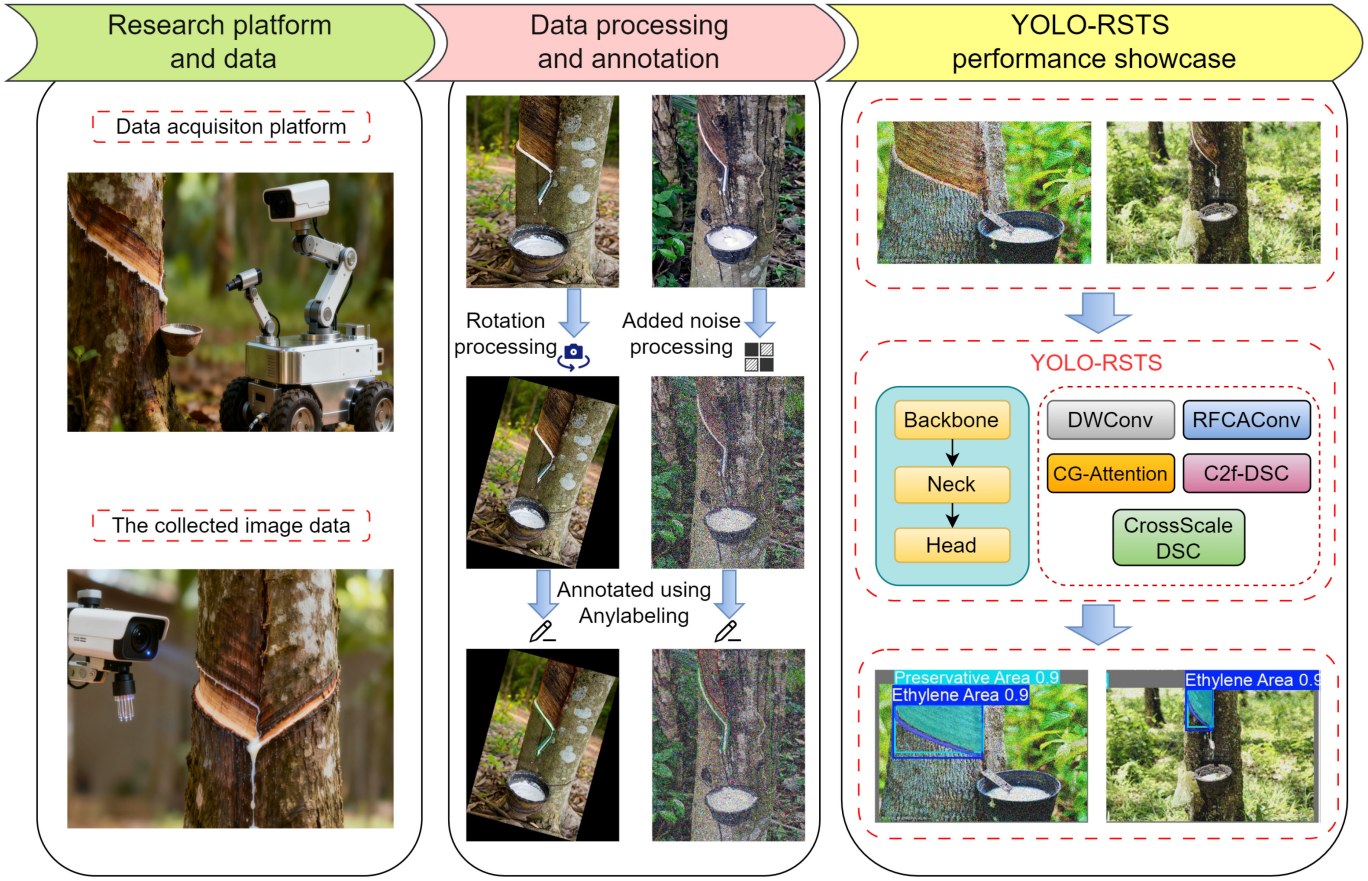
The complete methodological process of this study.

**Figure 1** presents the overall workflow of this research, which integrates dataset construction, annotation, and segmentation modeling for rubber tree spraying areas. The process begins with the acquisition of high-resolution visible light images, followed by preprocessing operations such as denoising, augmentation, and rotation to enhance image diversity and model generalization. The refined images are then annotated using the AnyLabeling tool, where stimulant and preservative regions on rubber tree trunks are carefully delineated to ensure precise and consistent ground-truth labels. These annotated samples serve as the foundation for training the proposed YOLO-RSTS segmentation model—an improved architecture built upon YOLOv12n-Seg, customized for the accurate extraction of spraying regions. After model training and optimization, YOLO-RSTS generates fine-grained segmentation maps that clearly differentiate the two target areas, enabling accurate recognition and analysis within real plantation environments.

The original YOLOv12n segmentation model serves as the foundation of this study. It features an efficient architecture that balances detection accuracy and computational cost, incorporating modules such as C2f, C3k2, and Bottleneck to enhance feature representation and model stability. The model also integrates attention mechanisms to improve its ability to capture key details and small targets in complex environments. Overall, YOLOv12 provides a robust and flexible baseline for segmentation tasks, upon which this research builds to further optimize performance in rubber tree spraying area detection.

**Figure 2:**
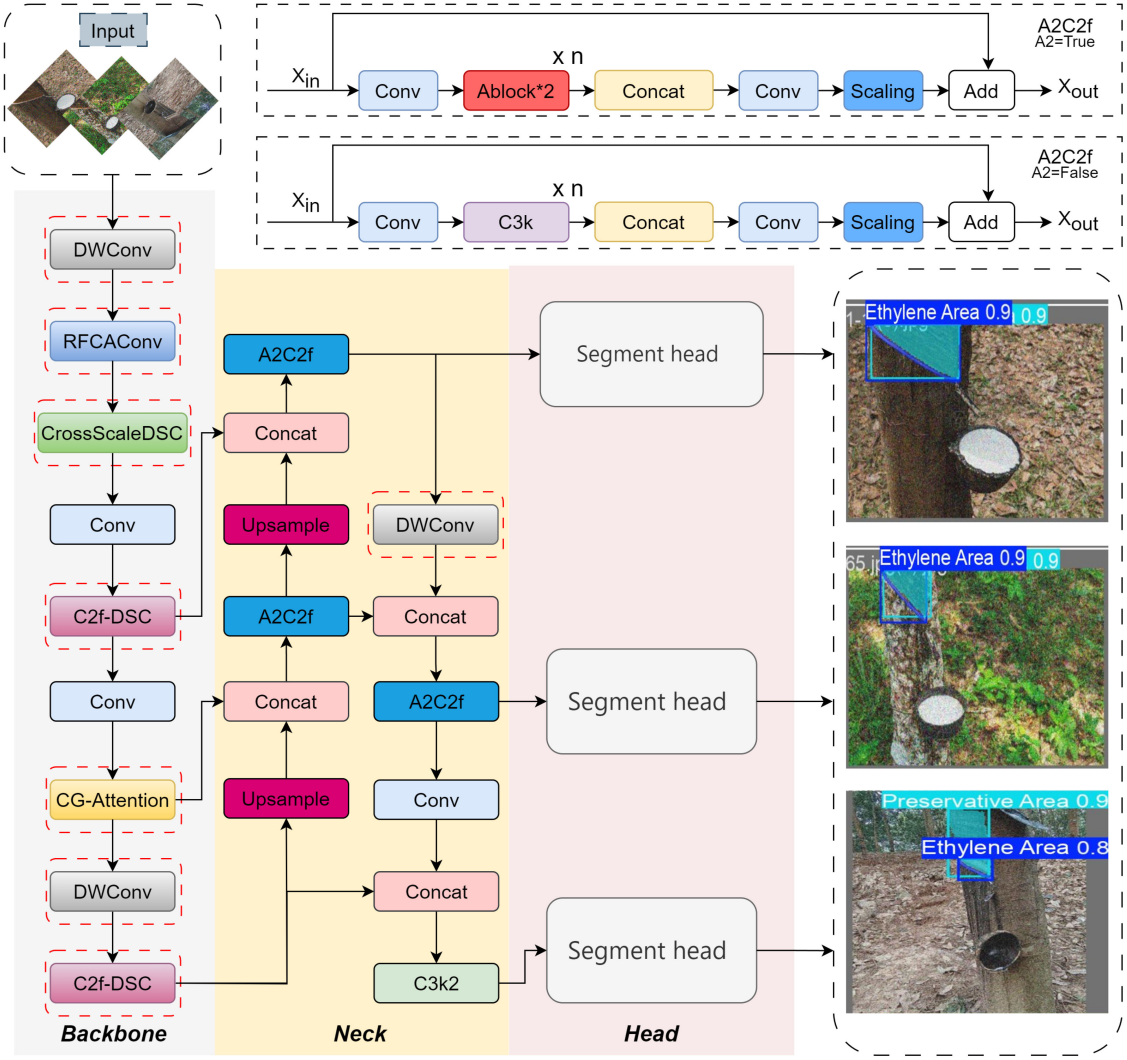
Complete structural diagram of the YOLO-RSTS segmentation model.

Building on the YOLOv12n-Seg framework, this study develops an enhanced segmentation architecture named YOLO-RSTS, tailored to the specific requirements of rubber tree spraying area detection. As illustrated in **Figure 2**, several targeted modifications are introduced within the backbone and neck to improve feature representation and computational efficiency. In the backbone, conventional convolutional layers are replaced with RFCAConv in the first and seventh positions, combining standard convolution with adaptive feature recalibration to capture more detailed texture information. The CrossScaleDSC and CG-Attention modules are integrated into the second and sixth layers, respectively, strengthening multi-scale feature fusion and global context modeling. Furthermore, the C2f-DSC module is embedded in the fourth and eighth layers, enhancing fine-grained feature interaction and contextual reasoning. To reduce model complexity, DWConv (depthwise separable convolution) substitutes standard convolutions in the zeroth backbone layer and the sixth neck layer, effectively lowering computational cost while maintaining segmentation precision. It is important to note that layer numbering starts from zero, meaning the first layer of the backbone is layer 0, the seventh layer is layer 6, and so on. With these architectural refinements, YOLO-RSTS achieves improved feature extraction capability, better adaptability to complex textures and lighting variations, and superior segmentation accuracy in the precise segmentation of rubber tree spraying areas.

### 2.1 Innovative CrossScaleDSC module

**Figure 3:**
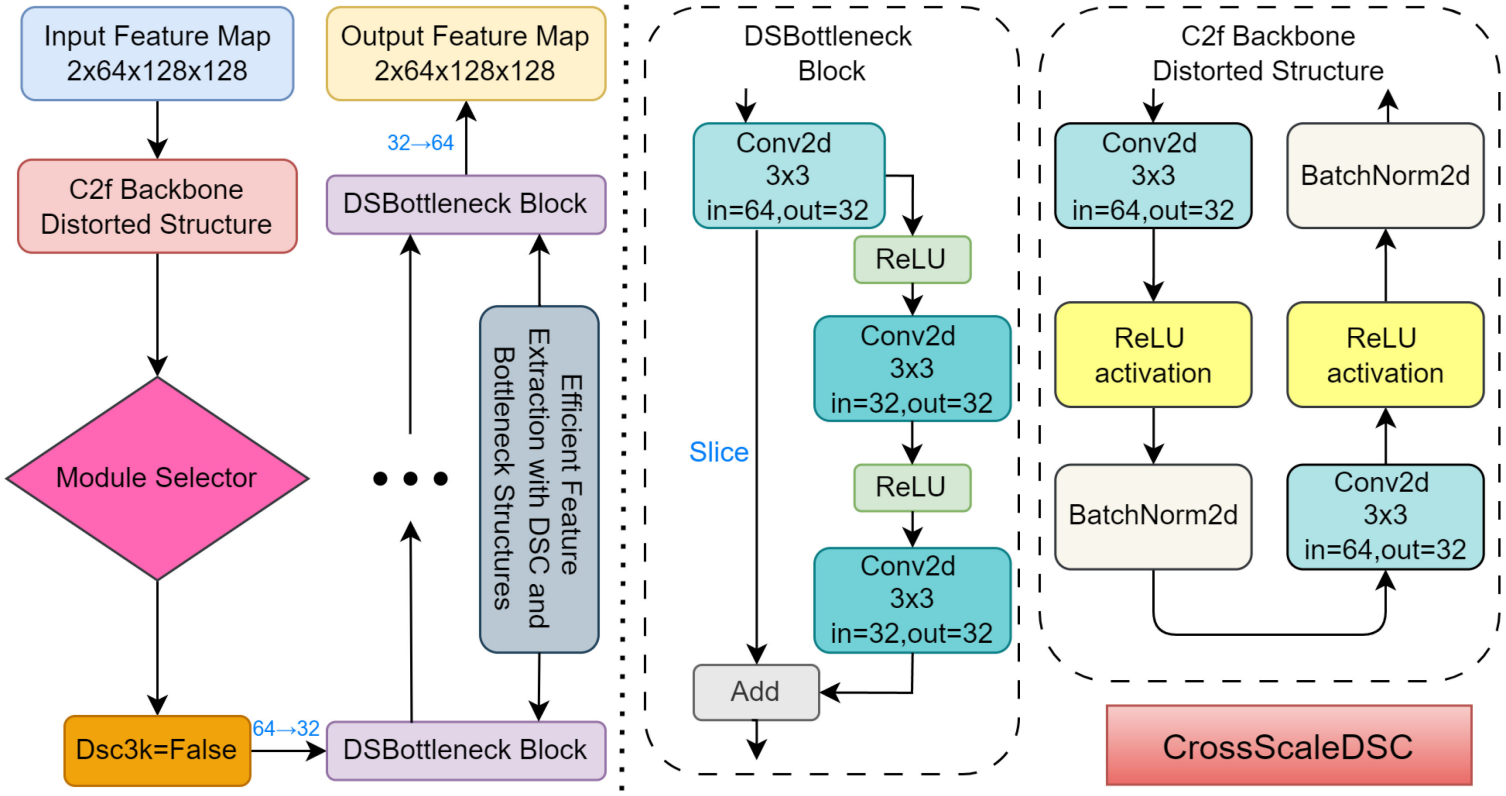
The internal construction of the CrossScaleDSC module.

The CrossScaleDSC (Cross-Scale Depthwise Separable Convolution) module was designed to address the specific challenges of rubber spraying area segmentation, where precise detection of small and complex regions is critical. Traditional convolutional layers, while effective, can be computationally expensive due to the large number of parameters they involve, creating challenges in both accuracy and efficiency during segmentation. To overcome this, the CrossScaleDSC module introduces key innovations: Depthwise Separable Convolutions (DSC), Bottleneck structures, and Shortcut Connections. DSC reduces computational complexity by separating convolution into depthwise and pointwise operations, improving efficiency without sacrificing accuracy. The Bottleneck structure optimizes feature extraction by reducing unnecessary dimensionality, while Shortcut Connections ensure that important low-level features are preserved, promoting smoother gradient flow and better feature retention. These innovations allow the module to effectively tackle the complexities of segmenting rubber spraying areas with high precision and efficiency. The detailed structure of the module is shown in the **Figure 3**.

The module begins by receiving an input tensor *x* from the preceding layer, which consists of feature maps with specific dimensions. The core of the CrossScaleDSC module is based on Depthwise Separable Convolution (DSC), which divides the traditional convolution operation into two distinct stages: depthwise convolution and pointwise convolution. The depthwise convolution applies a separate filter to each input channel, which allows the network to process each channel independently. This step reduces the number of parameters significantly compared to traditional convolutional layers. The output of the depthwise convolution is then passed through a pointwise convolution (1x1 convolution), which combines the depthwise-convolved features across all channels. Mathematically, the operations are as follows:


$$\text{Depthwise Conv}(x)=\sum_{i=1}^{C_{\text{in}}}{\text{Filter}}_{i}\;*\;x_{i}$$

$$\text{Pointwise Conv}(x)=\sum_{i=1}^{C_{\text{in}}}{\text{Filter}}_{i}^{'}\;*\;x_{i}$$

where $C_{\text{in}}$ is the number of input channels, xi is the feature map of the i-th channel, and ${\text{Filter}}_{i}$ and ${\text{Filter}}_{i}^{'}$ represent the depthwise and pointwise convolution filters, respectively. This separation ensures that the module processes the features efficiently without losing critical information.

Next, after the initial convolution steps, the module applies a **Bottleneck** structure. The bottleneck layer serves to reduce the dimensionality of the feature maps before expanding them back. This compression expansion strategy is designed to focus on the most important features while reducing computational load. The bottleneck layer can be represented as:


$$\text{Bottleneck}(x)=\text{Conv}(x,C_{\text{bottleneck}})\to \text{Conv}(x,C_{\text{out}})$$

Here, $C_{\text{bottleneck}}$ is the number of intermediate channels, typically smaller than the input channels $C_{\text{in}}$, and $C_{\text{out}}$ is the final output dimension of the convolution. This bottleneck structure helps in focusingthe network’s capacity on the most relevant features, optimizing both computational efficiency and performance.

The final step in the CrossScaleDSC module involves Shortcut Connections. These residual connections allow the input *x* to bypass some convolution operations and be directly added to the output of the module. This addition helps preserve important low-level features and facilitates more effective gradient flow through the network, addressing the issue of vanishing gradients often encountered in deeper networks. The formula for this operation is:


Output(x)=Conv(x)+x

where *x* is the input feature map, and Conv(*x*) is the output of the convolution operation. The addition of the identity mapping ensures that the model retains critical information while learning higher-level abstractions.

In the forward pass, the input tensor *x* flows through the CrossScaleDSC module, where it is first processed by the depthwise separable convolutions, followed by the bottleneck layer, and then passed through the shortcut connections. The final output is a refined feature map that captures essential high-level features while maintaining computational efficiency. By stacking multiple blocks of the CrossScaleDSC module, the network learns progressively more complex features as the data passes through each layer.

In summary, the CrossScaleDSC module combines three key innovations: Depthwise Separable Convolutions, Bottleneck Structures, and Shortcut Connections. These elements work together to create an efficient and powerful feature extraction block specifically designed for segmentation tasks. These design choices allow the CrossScaleDSC module to effectively address the challenges of segmenting small, intricate regions, such as those found in rubber spraying areas, while maintaining high precision and minimizing computational overhead.

### 2.2 Innovative CG-Attention module

**Figure 4:**
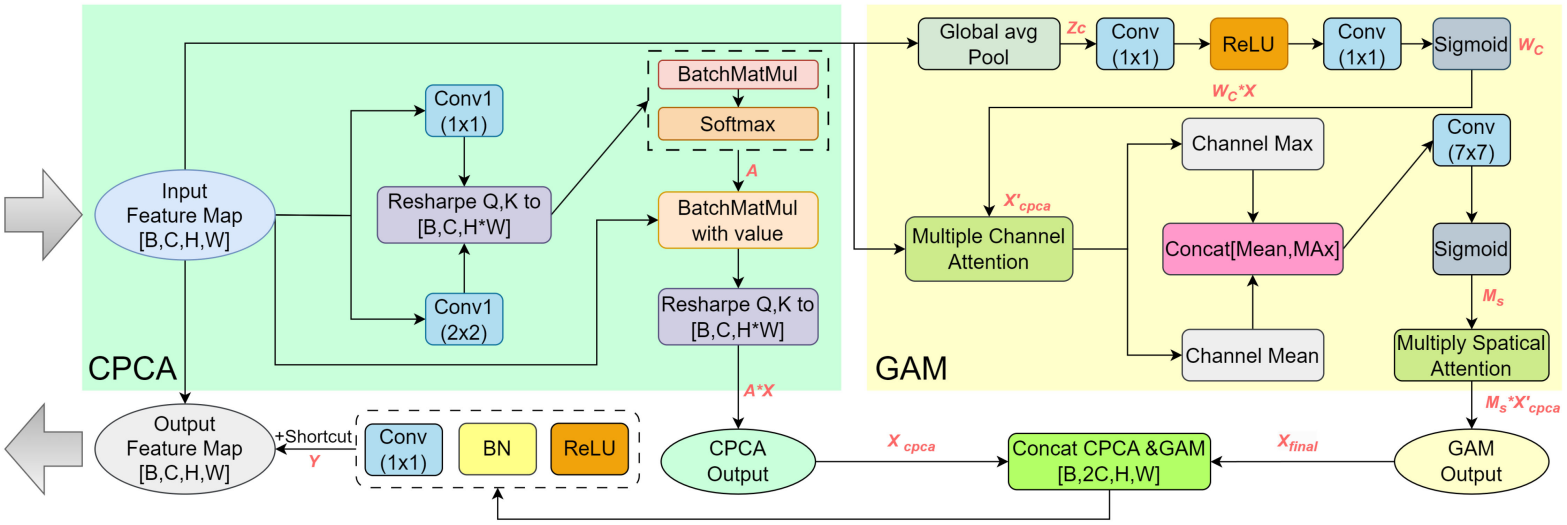
The internal construction of the CG-Attention module.

The CG-Attention module is developed to enhance the model’s ability to combine global semantic context with local fine-grained details, which is a critical challenge in segmentation tasks under complex conditions such as rubber spraying area detection. Traditional networks often struggle to balance these two aspects, resulting in inaccurate segmentation of areas with irregular textures or subtle boundaries. To address this issue, CG-Attention integrates Channel Principal Component Analysis Attention (CPCA) and Global Attention Mechanism (GAM), aiming to improve the model’s expressive power by enhancing both spatial and channel information in feature maps. The processing can be divided into two main parts: the CPCA part and the GAM part, which fuse information and enhance features through appropriate convolutional layers, activation functions, and attention mechanisms. The internal structure of the CG-Attention module is shown in **Figure 4**.

In the CPCA section, the input feature map $X\in R^{B\times C\times H\times W}$ is first processed using two convolution kernels with different sizes: a 1×1 convolution and a 2×2 convolution. These operations capture the relationships between channels and local region features, respectively. The query ( Q) and key ( K) tensors are then reshaped to [B,  C,  H×W] and passed through a BatchMatMul operation to calculate the correlation between channels. The results are subsequently processed using the Softmax function to generate the attention weights A between channels:


$$\text{A}=\text{Softmax}(\text{Q}{\text{K}}^{T})$$

This attention matrix allows the model to dynamically adjust the importance of each channel based on their relative relationships, enhancing the extraction of useful information. Finally, the attention weights are applied to the input feature map, resulting in the enhanced feature map ${\text{X}}_{\text{CPCA}}$:


$${\text{X}}_{\text{CPCA}}=\text{A}\cdot \text{X}$$

In the GAM section, global average pooling (GAP) is first applied to extract the global information of the input feature ma The global features ${\text{z}}_{c}$ for each channel are computed as:


$${\text{z}}_{c}=\frac{1}{HW}\sum_{i=1}^{H}\sum_{j=1}^{W}{\text{X}}_{c}(i,j)$$

These global features are then processed through a series of convolution operations (such as 1×1 convolutions) and activation functions (ReLU and Sigmoid), generating the attention weights ${\text{w}}_{c}$ for each channel. Simultaneously, the spatial attention map ${\text{M}}_{s}$ is computed by concatenating the max-pooling and average-pooling features, which are then processed by a convolutional layer:


$${\text{M}}_{s}=\sigma \;({\text{Conv}}_{7\times 7}(\text{MaxPool}(\text{X}),\text{AvgPool}(\text{X})))$$

This process helps the model focus on relevant spatial regions by leveraging both global and local feature information.

Finally, the channel and spatial attention are effectively combined. The channel attention is applied to the feature map as follows:


$${\text{X}}_{\text{CPCA}}^{'}={\text{w}}_{c}⊙\text{X}$$

where ⊙ denotes element-wise multiplication. The spatial attention map ${\text{M}}_{s}$ is then applied to the weighted feature map:


$${\text{X}}_{\text{final}}={\text{M}}_{s}⊙X_{\text{CPCA}}^{'}$$

Ultimately, the concatenated feature maps are processed through a 1 × 1 convolution layer, followed by batch normalization (BN) and ReLU activation, resulting in the final output feature map Y:


$$\text{Y}=\text{ReLU}(\text{BN}({\text{Conv}}_{1\times 1}([{\text{X}}_{\text{CPCA}},{\text{X}}_{\text{final}}])))$$

The effectiveness of the CG-Attention module arises from its ability to decompose and reweight feature representations along two complementary dimensions—global semantic context and fine local structures—and then integrate them through a parallel–serial coupling strategy. Specifically, CPCA focuses on channel-level redundancy and discriminability. By modeling inter-channel dependencies and applying a PCA-inspired projection, it suppresses noisy or weakly relevant channels and amplifies the most informative ones, thereby enhancing discriminative cues related to elongated boundaries and subtle textural variations characteristic of rubber spraying regions. In contrast, GAM enriches spatial reasoning by combining global average pooling, which captures long-range contextual dependencies along the trunk, with spatial attention that highlights localized boundary fluctuations. This dual mechanism enables GAM to refine spatial saliency while maintaining global structural awareness.

The coupling strategy further maximizes their complementary strengths. In the parallel stage, CPCA and GAM extract channel and spatial attention maps independently, preventing mutual interference and retaining their distinct representational benefits. In the serial stage, GAM subsequently refines the CPCA-enhanced features, yielding representations that maintain global consistency while being highly responsive to localized geometric and textural variations. This hierarchical refinement process allows the module to better handle anisotropic structures, irregular contours, and complex background patterns commonly present in rubber spraying areas, leading to more stable and accurate segmentation performance across diverse field scenarios.

Subsequent ablation experiments also validate that the coupling strategy of CPCA and GAM offers significant advantages over traditional attention mechanisms, particularly in handling fine-grained local features and global context, demonstrating superior robustness in addressing issues such as the aspect ratio and texture variations of rubber tree spraying areas. As a result, this optimization enables the CG-Attention module to provide a substantial performance boost.

### 2.3 Innovative C2f-DSC module

**Figure 5:**
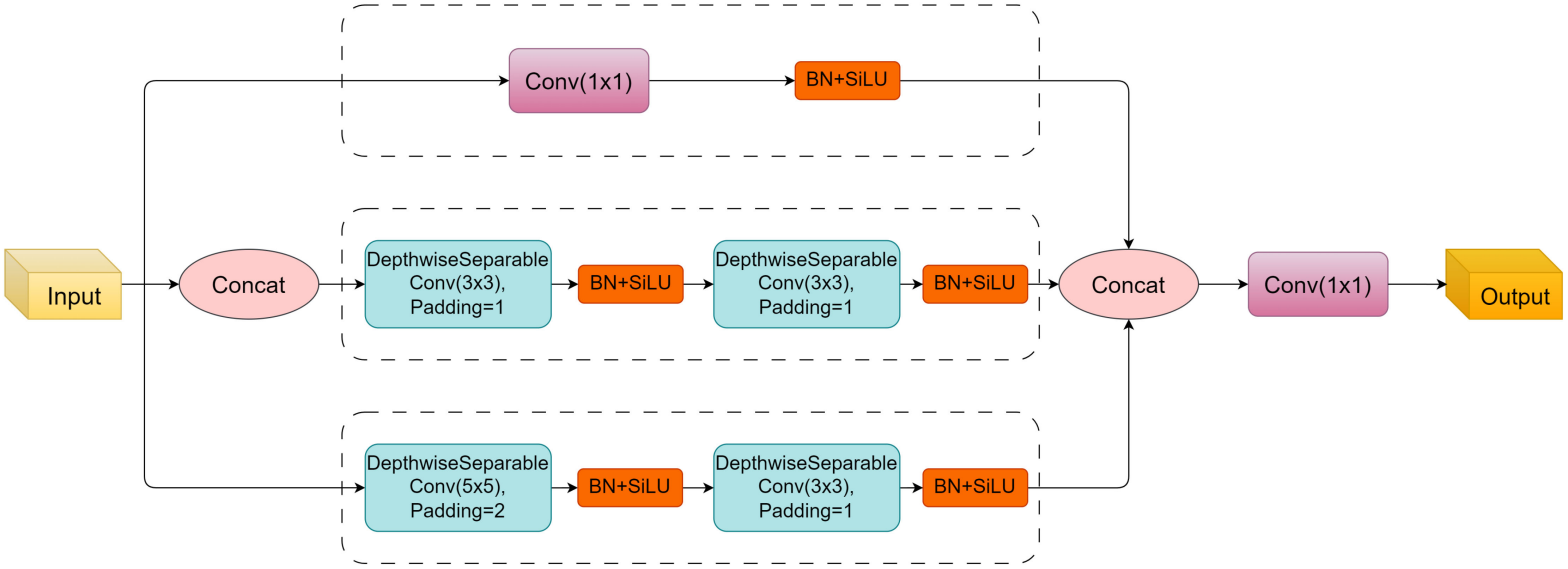
The internal construction of the C2f-DSC module.

Traditional models often face challenges when dealing with varying scales and intricate textures. To address this, C2f-DSC introduces a multi-path design that integrates depthwise separable convolutions with a lightweight convolution branch, allowing it to capture both global and local features effectively. This dual approach balances the extraction of detailed multi-scale information while reducing computational overhead. Additionally, the inclusion of bottleneck structures streamlines feature dimensionality, ensuring efficient processing without losing essential details. These innovations make C2f-DSC particularly effective for segmentation tasks involving diverse and complex backgrounds. The internal structure diagram of the C2f-DSC module is shown in the **Figure 5**.

The module receives an input tensor $X\in R^{B\times C_{\text{in}}\times H\times W}$ from the previous layer, where B is the batch size, $C_{\text{in}}$ is the number of input channels, and H×W are the spatial dimensions. To maintain computational efficiency while preserving sufficient local spatial information and global semantics, the C2f-DSC module is designed with three parallel paths. The first path is a lightweight 1 × 1 convolution branch, which serves to preserve and transform the original semantic information. The second and third paths are depthwise separable convolution (DSC) branches, each consisting of two consecutive 3 × 3 DSC units, aimed at extracting spatial features and reducing computational complexity.

In these three parallel paths, the first path applies a simple 1 × 1 convolution to perform a linear transformation on the input features, expressed as:


$$U=\text{BN}({\text{Conv}}_{1\times 1}(X)),$$

where BN denotes the BatchNorm operation, which helps accelerate training and stabilize the learning process. The purpose of this path is to retain the global semantic information of the input and map it to a specific feature space.

The second and third paths use depthwise separable convolutions (DSC) to extract local spatial information. Each DSC unit consists of a depthwise convolution followed by a pointwise convolution, as described by the formula:


$$\text{DSC}(X)={\text{Conv}}_{1\times 1}({\text{Conv}}_{3\times 3}^{\text{dw}}(X)),$$

where ${\text{Conv}}_{3\times 3}^{\text{dw}}$ represents the depthwise convolution, which operates independently on each channel, and Conv_1×1_ is the pointwise convolution, used for channel mixing. The advantage of depthwise separable convolutions is that they decompose the traditional convolution operation into two more lightweight steps, thereby significantly reducing both the parameter count and computational cost.

Next, the outputs of the three paths are concatenated along the channel dimension to form a higher dimensional feature tensor Z. The concatenated feature map is then passed through a 1 × 1 convolution for channel fusion, resulting in the final fused representation $\hat{Z}$, expressed as:


$$\hat{Z}=\text{SiLU }(\text{BN }({\text{Conv}}_{1\times 1}(Z))),$$

where SiLU is the Sigmoid Linear Unit (SiLU) activation function, which enhances non-linear expressiveness. After this step, the module effectively fuses the features extracted from different branches while avoiding redundant information accumulation.

Finally, the output Y is obtained by applying another 1 × 1 convolution to the fused feature map $\hat{Z}$ for channel mapping, resulting in the output tensor of shape $R^{B\times C_{\text{out}}\times H\times W}$:


$$Y=\text{BN }({\text{Conv}}_{1\times 1}(\hat{Z})).$$

When the output channel number $C_{\text{out}}$ does not match the input channel number $C_{\text{in}}$, a 1×1 projection operation can be applied to the input to align the dimensions and the residual connection is added to the final output:


$$Y_{\text{out}}=Y+{\text{Proj}}_{1\times 1}(X).$$

The C2f-DSC module addresses the fundamental limitations of traditional convolutions by introducing a structured decomposition of feature learning. Depthwise separable convolutions perform an implicit low-rank factorization of the convolution kernel, separating spatial filtering from channel mixing. This decoupling not only reduces computational redundancy but also strengthens the model’s inductive bias toward capturing directional and fine-scale structures—properties that closely match the geometry of rubber spraying regions, which often contain elongated boundaries and weak, anisotropic textures. The multi-path architecture further enriches this representational space by allocating complementary roles to different branches: the 1×1 convolution path aggregates global semantic context, while the two DSC paths extract localized, orientation-sensitive features. Together, these parallel branches expand the expressive capacity of the feature space without increasing computational burden, enabling the network to model irregular structures more precisely than standard convolutional blocks.

Beyond architectural decomposition, the C2f-DSC module benefits from a theoretically grounded feature fusion mechanism and stability-oriented optimization design. The fusion stage—implemented through a learnable 1×1 projection—acts as a unifying operator that aligns multi-scale responses into a coherent latent space, similar to basis aggregation in multi-resolution signal analysis. This enables the network to combine broad contextual cues with high-frequency boundary information in a balanced manner. Batch Normalization stabilizes the joint optimization of parallel paths by reducing distributional shifts, while the SiLU activation contributes smoother gradient dynamics and improved sensitivity to subtle intensity transitions. These properties are particularly important in segmenting fine, low-contrast spraying regions in complex field environments. Collectively, these mechanisms form an integrated framework that enhances both expressiveness and optimization robustness, yielding the superior fine-grained segmentation accuracy observed in the ablation studies.

## 3 Datasets and evaluation indicators

### 3.1 Data acquisition and dataset construction

The training and validation sets used in this study were constructed by integrating two complementary image sources to form an optical dataset for rubber spraying-region segmentation. One component comprises publicly available plantation photos harvested from online repositories, bringing broad geographic and visual variability. The other component consists of carefully collected forest images from a plantation in Xishuangbanna, Yunnan, taken in April 2025 under clear, well-lit conditions to provide high-quality, controlled examples. Merging these web-sourced and field-captured images yields a dataset that balances diversity with consistency, improving the model’s ability to generalize across different plantations and environmental settings.

To improve dataset robustness and broaden training variability, we applied a set of augmentation techniques, including random rotations, synthetic noise injection and conversion to grayscale, which increase appearance diversity and help the model generalize to varied field conditions. The annotated dataset is organized into two classes: catalyst spraying areas and preservative spraying areas. This distinction corresponds to their different agronomic functions: catalysts are applied to stimulate latex flow and increase yield, while preservatives are used after tapping to protect the exposed tree surface from oxidation and decay.

The dataset comprises a total of 1,306 images, with 1,107 images allocated for training and 199 images for validation, ensuring sufficient support for both model training and evaluation. The image annotation was performed using the AnyLabelling software tool, ensuring accuracy and consistency in marking the spraying areas within each image. Overall, this dataset, combining images from diverse sources, enhanced through various data augmentation techniques, and meticulously annotated, is well-suited for the precise segmentation of rubber spraying areas. It provides valuable data support for the intelligent management of rubber plantations and serves as an invaluable resource for research in related fields.

Moreover, to better reflect the variability encountered in real operational environments, we conducted field data collection on December 2, 2025, in Danzhou City, Hainan Province. A total of 346 raw images were acquired using two consumer-grade mobile devices (Huawei Mate 60 and Xiaomi 14) to serve as the test set for this study. The sampling covered representative rubber plantation sites across Hainan, including Lingao County (County Road 307, 97.0 m elevation, overcast, 22 °C; National Road 225, 97.7 m elevation, overcast, 21 °C; County Road 307, 95.6 m elevation, overcast, 22 °C) and Danzhou City (Beiya, 135.9 m elevation, cloudy, 26 °C; Fanbao, 140.2 m elevation, cloudy, 26 °C; Jianling, 172.8 m elevation, cloudy, 26 °C; Route 016, 116.4 m elevation, overcast, 24 °C; Shiwucun, 112.5 m elevation, overcast, 25 °C). The environmental temperature ranged from 21 °C to 26 °C, with predominantly cloudy or overcast conditions, reflecting the typical low-light, low-contrast, and high-humidity characteristics of winter rubber plantations in Hainan.

All images were captured at a resolution of 1200 × 1600 pixels (96 dpi), encompassing diverse imaging perspectives, including varying observation distances (long-range and close-range), different shooting heights (high and low viewpoints), and multiple view angles (frontal, lateral, and oblique). Compared with images acquired in the spring season in Xishuangbanna, the winter conditions in Hainan introduce notable differences in vegetation structure, tree morphology, illumination geometry, and background texture complexity, thereby increasing the difficulty of detection and region segmentation tasks.

Overall, this test set provides strong representativeness in geographic diversity, seasonal variation, phenological differences, and device heterogeneity, making it a rigorous benchmark for assessing model generalization under OOD conditions. The subsequent experiments show that this cross-regional dataset effectively validates the robustness and applicability of the improved YOLO-RSTS model, offering reliable evidence of its capability to operate in diverse and complex rubber plantation environments.

### 3.2 Evaluation indicators

This study employs five metrics to evaluate detection accuracy and deployment efficiency: Recall, AP/mAP, F1 score, GFLOPs and Parameters. The former three quantify detection quality, while the latter two reflect inference cost and model size, as defined in **Equations 1**–**6**.

(1)Recall: proportion of true positives that are correctly detected.

(1) $$\text{Recall }=\;\frac{\text{TP}}{\text{TP}+\text{FN}}$$

(2)Average Precision (AP) and mean AP (mAP): AP is the area under the precision–recall curve for a class. mAP is the arithmetic mean of AP over *K* classes.

(2) $$AP=\int_{0}^{1}P(r)\text{d}r,$$

(3) $$\text{mAP}=\frac{1}{K}\sum_{i=1}^{K}AP_{i},$$

where *P*(*r*) denotes precision as a function of recall *r*.

(3)F1 score: the harmonic mean of precision and recall.

(4) $$F_{1}\;=\;2\cdot \frac{\text{Precision}\times \text{Recall}}{\text{Precision}+\text{Recall}}$$

(4)GFLOPs: total floating-point operations per inference, expressed in billions.

(5) $$\text{GFLOPs }=\;\frac{\text{Total floating}-\text{point operations per inference}}{10^{9}}$$

(5)Parameters: total number of trainable weights. For a standard convolutional layer with optional bias, the parameter count is:

(6) $$\text{Parameters }=\;(C_{\text{in}}\cdot K_{h}\cdot K_{w})\cdot C_{\text{out}}\;+\;C_{\text{out}}$$

where $C_{\text{in}}$ and $C_{\text{out}}$ are the input and output channels, and $K_{h},K_{w}$ are kernel height and width.

These metrics jointly assess detection effectiveness (Recall, AP/mAP, F1) and deployment feasibility (GFLOPs, Parameters), facilitating comparisons between accuracy and computational cost.

## 4 Experiments and result

To comprehensively evaluate the performance of the proposed YOLO-RSTS model, this study designed and implemented two types of experiments: ablation experiments and model comparison experiments.

In the ablation experiments, not only the innovative modules but also all modified components of the model were subjected to detailed ablation testing. Key modules were sequentially removed or replaced to assess their specific contributions to the overall performance improvement. Additionally, we compared CG-Attention with other popular feature fusion module. To ensure fairness and consistency, identical experimental settings and evaluation standards were adopted.

In the model comparison experiments, YOLO-RSTS was compared vertically with other models in the YOLO series (such as YOLOv8, YOLOv10, YOLOv11, etc.) to comprehensively assess the extent of performance improvement in YOLO-RSTS compared to different versions of the YOLO model. To ensure fairness, the experimental design standardized the input data and evaluation criteria for each model, ensuring that both vertical and horizontal comparisons were conducted under the same conditions.

These experiments are designed to quantify the specific contributions of each novel component to model performance and to clarify YOLO-RSTS’s superior performance, thereby providing valuable insights for future related research.

### 4.1 Configuration of experimental environment

All segmentation models were trained and tested on two NVIDIA Tesla P100 GPUs with 16 GB memory each. The experiments ran in a Python 3.11 environment using PyTorch 2.2 compiled with CUDA 11, and FlashAttention v2.7.3 was installed from the official precompiled wheel to accelerate attention layers. Input images were resized to 640 × 640, the training batch size was 64, and each model was trained for 250 epochs with the AdamW optimizer. The initial learning rate was 0.01 and followed a cosine decay schedule, momentum was set to 0.937 and weight decay to 5e-4. Data augmentation consisted of Mosaic (p = 1.0) and random horizontal flipping (p = 0.5). Each training checkpoint and log are eventually archived for later evaluation and comparison.

### 4.2 Ablation experiments

#### 4.2.1 Ablation experiments on the dataset

To evaluate the effectiveness and specific contributions of the individual enhancements in YOLO-RSTS and their various combinations, this study conducted a series of ablation experiments. **Table 1** summarizes the results of the ablation experiments. The experiments examined both the contribution of each individual module to the overall performance and the impact of several partial module combinations on segmentation effectiveness, with all variants compared to the full YOLO-RSTS model. The results clearly demonstrate that each individual module significantly improves spray-area segmentation accuracy, while these combinations still show a certain degree of performance gap compared to the complete YOLO-RSTS model. These findings indicate that the proposed independent enhancements exhibit important synergistic effects rather than simple interchangeability.

**Table 1:** Ablation experiments on the dataset.

Model	Precision	Recall	mAP0.50	mAP0.50:0.95	F1 score	GFLOPs (B)	Param (M)
YOLOv12n	0.819(↑ 6.3%)	0.740(↑ 8.1%)	0.788(↑ 6.3%)	0.424(↑ 2.7%)	0.777(↑ 7.3%)	7.0	2.72(↓ 14.3%)
YOLOv12n+RFCAConv	0.905	0.727(↑ 9.4%)	0.809(↑ 4.2%)	0.419(↑ 3.2%)	0.806(↑ 4.4%)	7.2	2.74(↓ 15.0%)
YOLOv12n+DWConv	0.806(↑ 7.6%)	0.760(↑ 6.1%)	0.792(↑ 5.9%)	0.423(↑ 2.8%)	0.782(↑ 6.8%)	6.9	2.69(↓ 13.4%)
YOLOv12n+CrossScaleDSC	0.846(↑ 3.6%)	0.731(↑ 9.0%)	0.798(↑ 5.3%)	0.434(↑ 1.7%)	0.784(↑ 6.4%)	7.6	2.75(↓ 15.3%)
YOLOv12n+CG-Attention	0.886	0.760(↑ 6.1%)	0.815(↑ 3.6%)	0.445(↑ 0.6%)	0.818(↑ 3.2%)	6.7	2.64(↓ 11.7%)
YOLOv12n+C2f-DSC	0.808(↑ 7.4%)	0.779(↑ 4.2%)	0.806(↑ 4.5%)	0.442(↑ 0.9%)	0.793(↑ 5.8%)	7.2	2.54(↓ 8.3%)
YOLOv12n+RFCAConv+DWConv	0.862(↑ 2.0%)	0.777(↑ 4.4%)	0.842(↑ 0.9%)	0.427(↑ 2.4%)	0.817(↑ 3.3%)	7.1	2.71(↓ 14.0%)
YOLOv12n+RFCAConv+CrossScaleDSC	0.853(↑ 2.9%)	0.828	0.828(↑ 2.3%)	0.460	0.840(↑ 1.0%)	7.8	2.77(↓ 15.9%)
YOLOv12n+RFCAConv+CG-Attention	0.808(↑ 7.40%)	0.763(↑ 5.8%)	0.819(↑ 3.2%)	0.448(↑ 0.3%)	0.785(↑ 6.6%)	6.9	2.66(↓ 12.4%)
YOLOv12n+RFCAConv+C2f-DSC	0.831(↑ 5.1%)	0.791(↑ 3.0%)	0.813(↑ 3.8%)	0.451	0.811(↑ 4.0%)	7.3	2.56(↓ 9.0%)
YOLOv12n+Manba-DSC+CG-Attention	0.822(↑ 6.0%)	0.769(↑ 5.2%)	0.798(↑ 5.3%)	0.425(↑ 2.6%)	0.795(↑ 5.6%)	6.7	2.64(↓ 11.7%)
YOLOv12n+Manba-DSC+C2f-DSC	0.819(↑ 6.3%)	0.733(↑ 8.8%)	0.785(↑ 6.6%)	0.437(↑ 1.4%)	0.774(↑ 7.7%)	7.2	2.55(↓ 8.6%)
YOLOv12n+CG-Attention+C2f-DSC	0.848(↑ 3.4%)	0.746(↑ 7.5%)	0.796(↑ 5.5%)	0.442(↑ 0.9%)	0.794(↑ 5.7%)	6.9	2.57(↓ 9.3%)
YOLOv12n+RFCAConv+DWConv+CrossScaleDSC	0.849(↑ 3.3%)	0.743(↑ 7.8%)	0.810(↑ 4.1%)	0.438(↑ 1.3%)	0.792(↑ 5.8%)	7.7	2.73(↓ 14.7%)
YOLOv12n+RFCAConv+DWConv+CG-Attention	0.870(↑ 1.2%)	0.754(↑ 6.70%)	0.814(↑ 3.7%)	0.426(↑ 2.5%)	0.808(↑ 4.3%)	6.8	2.63(↓ 11.4%)
YOLOv12n+RFCAConv+DWConv+C2f-DSC	0.792(↑ 9.0%)	0.771(↑ 5.0%)	0.798(↑ 5.3%)	0.440(↑ 1.1%)	0.781(↑ 6.9%)	7.2	2.53(↓ 7.9%)
YOLOv12n+RFCAConv+CrossScaleDSC+CG-Attention	0.862(↑ 2.0%)	0.737(↑ 8.4%)	0.799(↑ 5.2%)	0.432(↑ 1.9%)	0.795(↑ 5.6%)	7.2	2.63(↓ 11.4%)
YOLOv12n+DWConv+CrossScaleDSC+C2f-DSC	0.832(↑ 5.0%)	0.801(↑ 2.0%)	0.820(↑ 3.1%)	0.443(↑ 0.8%)	0.816(↑ 3.4%)	7.7	2.53(↓ 7.9%)
YOLOv12n+CrossScaleDSC+CG-Attention+C2f-DSC	0.819(↑ 6.3%)	0.765(↑ 5.6%)	0.789(↑ 6.2%)	0.445(↑ 0.6%)	0.791(↑ 5.9%)	7.5	2.49(↓ 6.4%)
YOLOv12n+RFCAConv+DWConv+CrossScaleDSC+CG-Attention	0.843(↑ 3.9%)	0.744(↑ 7.7%)	0.816(↑ 3.5%)	0.445(↑ 0.6%)	0.790(↑ 6.0%)	7.4	2.65(↓ 12.1%)
**YOLO-RSTS(Ours)**	**0.882**	**0.821**	**0.851**	**0.451**	**0.850**	**7.0**	**2.33**

Bold values highlight the proposed YOLO-RSTS model, emphasizing its effectiveness and facilitating intuitive comparison.

From the experimental results, it is evident that the YOLO-RSTS model significantly improves overall performance in the rubber tree spraying area segmentation task through innovative structural design and module optimization. Compared to the baseline model, YOLOv12n, YOLO-RSTS excels across several key metrics, particularly achieving a 7.3% improvement in mAP0.50, demonstrating a substantial enhancement in region segmentation accuracy and effectively reducing both false positives and false negatives. Specifically, the RFCAConv and DWConv modules optimize the feature extraction process, enhancing the fine-grained recognition of the rubber tree spraying areas. The former improves precision by 6.3%, while the latter increases recall by 8.1%. These improvements allow the model to accurately identify target areas while minimizing missed detections, thereby further improving segmentation completeness. The innovative CrossScaleDSC and CG-Attention modules further optimize the convolution layers’ computations and feature expression capabilities. The former reduces computational complexity through lightweight convolution operations, while the latter enhances feature expression in complex backgrounds. Additionally, the C2f-DSC module strengthens multi-scale processing capabilities, ensuring the model remains robust and efficient when handling regions of various sizes. With the integration of these modules, YOLO-RSTS achieves a 7.5% improvement in F1 score compared to the YOLOv12n model, demonstrating a well-balanced trade-off between precision and recall.

It is worth noting that YOLO-RSTS successfully reduces parameter count (Param(M)) from 2.73 to 2.65, a 14.7% decrease, while optimizing computational resources. This indicates that despite the improvements in segmentation accuracy, the model still maintains a balance between computational efficiency and inference speed. The GFLOPs also remain unchanged, proving that the model continues to meet real-time application requirements without increasing computational demand. Moreover, in comparison with other combined models, YOLO-RSTS consistently outperforms in terms of accuracy and completeness. For example, compared to the YOLOv12n+RFCAConv+DWConv+CG-Attention combination, which is a suboptimal configuration, YOLO-RSTS demonstrates a higher performance, especially with a 1.7% higher F1 score. These results showcase YOLO-RSTS’ significant advantages in the rubber tree spraying area segmentation task, achieving the best performance.

The success of the YOLO-RSTS model lies in its deep structural design and the efficient integration of various modules. First, the RFCAConv and DWConv modules optimize the feature extraction process, enabling the model to capture more diverse features across different scales. By adjusting the layer structure of these two modules (replacing the YOLOv12n model’s 1st and 7th layers, and the 0th and 15th layers), the model excels in detail capturing, particularly for tasks like rubber tree spraying area segmentation, which require high localization precision. Next, the innovative CrossScaleDSC module is introduced, which uses a lightweight convolution layer design to significantly reduce computational complexity while enhancing the efficiency of information flow. This module offers particular advantages for small object detection. By replacing the 2nd layer, inference speed is optimized, ensuring high efficiency when processing the fine details of rubber tree features. The improved CG-Attention module further enhances the model’s ability to capture spatial information and improves segmentation accuracy in complex backgrounds. Specifically, by applying this module to optimize the 6th layer, the model’s adaptability to dynamic environments is increased, significantly reducing the occurrence of missed segmentations. Finally, the innovative C2f-DSC module strengthens multi-scale processing by optimizing the fusion of the 4th and 8th layers. This improves the model’s ability to handle both large and detailed areas, ensuring robust performance and high precision across different scales. Here, the 0th layer refers to the first layer when counting from the backbone network to the head network, starting from layer 0 of the backbone.

In summary, YOLO-RSTS, through its carefully designed modules and architectural optimizations, not only significantly improves performance metrics but also achieves a balance in computational resource consumption. This ensures its potential for deployment and application in real-world environments.

#### 4.2.2 Comparison of CG-Attention with other feature fusion module

To more comprehensively assess the performance improvements brought by the CG-Attention module, we compare it with other classic feature fusion modules, including RepC3, C3Ghost, C2f, and A2C2f. This comparison aims to highlight the advantages of CG-Attention in terms of segmentation accuracy and computational cost. Moreover, analysis of each module’s performance further clarifies the advantages of CG-Attention over other widely used fusion strategies. **Table 2** presents the comparison results.

**Table 2:** Comparison of CG-Attention with other feature fusion module on the dataset.

Model	Precision	Recall	mAP0.50	mAP0.50:0.95	F1 score	GFLOPs (B)	Param (M)
YOLOv12n	0.819(↑ 6.7%)	0.740(↑ 2.0%)	0.788(↑ 2.7%)	0.424(↑ 2.1%)	0.777(↑ 4.1%)	7.0	2.72(↓ 2.9%)
YOLOv12n+C2fCIB	0.841(↑ 4.5%)	0.626(↑ 13.4%)	0.659(↑ 15.6%)	0.359(↑ 8.6%)	0.718(↑ 10.0%)	7.4	2.73(↓ 3.3%)
YOLOv12n+RepC3	0.859(↑ 2.7%)	0.752(↑ 0.8%)	0.809(↑ 0.6%)	0.446	0.802(↑ 1.6%)	8.8	2.76(↓ 4.3%)
YOLOv12n+C2	0.872(↑ 1.4%)	0.744(↑ 1.6%)	0.803(↑ 1.2%)	0.444(↑ 0.1%)	0.803(↑ 1.5%)	8.0	2.74(↓ 3.6%)
YOLOv12n+C2f	0.842(↑ 4.4%)	0.774	0.814(↑ 0.1%)	0.461	0.807(↑ 1.2%)	8.5	2.75(↓ 4.0%)
YOLOv12n+C3x	0.809(↑ 7.7%)	0.781	0.805(↑ 1.0%)	0.437(↑ 0.8%)	0.795(↑ 2.3%)	7.3	2.73(↓ 3.3%)
YOLOv12n+C3	0.845(↑ 4.1%)	0.734(↑ 2.6%)	0.789(↑ 2.6%)	0.435(↑ 1.0%)	0.786(↑ 3.2%)	7.5	2.73(↓ 3.3%)
YOLOv12n+A2C2f	0.822(↑ 6.4%)	0.752(↑ 0.8%)	0.806(↑ 0.9%)	0.438(↑ 0.7%)	0.785(↑ 3.3%)	8.0	2.74(↓ 3.6%)
**YOLOv12n+CG-Attention (Ours)**	**0.886**	**0.760**	**0.815**	**0.445**	**0.818**	**6.7**	**2.64**

Bold values highlight the proposed YOLO-RSTS model, emphasizing its effectiveness and facilitating intuitive comparison.

Table results indicate that the CG-Attention achieves a superior trade-off between performance and resource consumption. Specifically, YOLOv12n+CG-Attention attains a precision of 0.886, recall of 0.760, and F1 score of 0.818; mAP0.50 is 0.815 and mAP0.50:0.95 is 0.445. This model also exhibits the lowest computational cost and parameter count (6.7 GFLOPs; 2.64 M parameters). Relative to the baseline YOLOv12n (precision 0.819, F1 0.777, 7.0 GFLOPs, 2.72 M parameters), precision increases by 6.7%, F1 score increases by 4.1%, and parameter count decreases by approximately 2.9%. Against other fusion alternatives, the differences are instructive: RepC3 yields a marginally higher mAP0.50:0.95 (0.446 vs. 0.445) but incurs substantially greater computational cost and parameters (8.8 GFLOPs, 2.76 M), indicating that the small mAP gain comes at a high resource cost; C2f attains higher recall (0.774) but lower Precision (0.842) and F1 score (0.807) and requires more compute/parameters (8.5 GFLOPs/2.75 M); C2fCIB performs inconsistently on this dataset (Recall = 0.626, mAP0.50 = 0.659), struggling to balance precision and recall; C3/C3x show strengths in recall or other local metrics (C3x Recall = 0.781) but lag in Precision and overall F1 score while also having higher resource footprints; A2C2f attains similar mAP and recall (mAP0.50 = 0.806, Recall = 0.752) yet requires more compute and parameters (8.0 GFLOPs, 2.74 M). Overall, CG-Attention provides a more robust and efficient precision–cost trade-off on this dataset. In summary, CG-Attention achieves higher precision and a more robust F1 score while maintaining the lowest computational and parameter cost, yielding a superior accuracy–cost trade-off compared with most baseline methods.

The performance gains of CG-Attention stem from three complementary architectural choices. First, a GAP→MLP channel prior followed by a channel-refinement mapping projects channel statistics into spatially-varying per-channel coefficients, enabling the importance of a given channel to adapt across spatial locations. Second, channel-level and spatial-level attentions are coupled via element-wise multiplication, so that informative channels are selected while salient pixels are simultaneously emphasized—this joint constraint concentrates responses at object boundaries and on small targets. Third, a lightweight shallow compensation branch injects low-level detail into the joint attention, mitigating the loss of boundary and texture information that can occur during high-level semantic reallocation. By contrast, common alternatives exhibit specific limitations tied to their internal designs. RepC3-style reparameterization and multi-branch schemes increase representational richness through parallel convolutions and denser channel mixing but typically demand larger intermediate feature maps and higher GFLOPs/parameter counts, and they do not provide position-varying per-channel modulation. C3/C3x (CSP/bottleneck-based channel partitioning and residual bottlenecks) focus on network depth and channel splitting to improve gradient flow and global representation, yet this partitioning tends to reduce continuous attention to fine-grained spatial patterns. Lightweight variants such as C3Ghost and C2fCIB save parameters via GhostConv or cheap feature synthesis but sacrifice feature diversity and fine-detail representation, harming precision and boundary preservation. Methods that rely primarily on global channel scaling or single-mode spatial attention (e.g., C2f, A2C2f) can improve semantic aggregation but lack mechanisms to decouple channel statistics and map them into spatially-varying per-channel weights, which limits performance in tasks requiring precise localization and discrimination of adjacent semantics. Consequently, CG-Attention’s targeted channel–position coupling + shallow compensation design addresses these shortcomings and delivers higher precision and a more robust F1 score while keeping computational and parameter overheads low.

### 4.3 Model comparison experiments

To further comprehensively evaluate the performance gains and advantages of YOLO-RSTS, this study conducted comparative experiments against several state-of-the-art YOLO variants (e.g., YOLO11, YOLOv13) using the same dataset and identical experimental settings. For variants with publicly available segmentation implementations, the official segmentation models were used; for variants without a segmentation release, segmentation versions were created by replacing their detection heads with the segmentation head, while the remainder of the network and the training hyperparameters were kept unchanged. The results of the segmentation model comparison are shown in the **Table 3**.

**Table 3:** Model comparison experiments on the dataset.

Model	Precision	Recall	mAP0.50	mAP0.50:0.95	F1 score	GFLOPs (B)	Param (M)
YOLOv8n	0.860(↑ 2.2%)	0.710(↑ 11.1%)	0.810(↑ 4.1%)	0.446(↑ 0.5%)	0.778(↑ 7.2%)	12.0(↓ 41.7%)	2.36(↓ 1.3%)
YOLOv10n	0.837(↑ 4.5%)	0.751(↑ 7.0%)	0.791(↑ 6.0%)	0.451	0.791(↑ 5.9%)	8.5(↓ 17.6%)	2.75(↓ 15.3%)
YOLO11n	0.794(↑ 8.8%)	0.785(↑ 3.6%)	0.805(↑ 4.6%)	0.447(↑ 0.4%)	0.789(↑ 6.1%)	10.2(↓ 31.4%)	2.84(↓ 17.9%)
YOLOv12n	0.819(↑ 6.3%)	0.740(↑ 8.1%)	0.788(↑ 6.3%)	0.424(↑ 2.7%)	0.777(↑ 7.3%)	7.0	2.72(↓ 14.3%)
YOLOv13n	0.821(↑ 6.1%)	0.703(↑ 11.8%)	0.776(↑ 7.5%)	0.409(↑ 4.2%)	0.758(↑ 9.2%)	10.1(↓ 30.7%)	2.70(↓ 13.7%)
**YOLO-RSTS (Ours)**	**0.882**	**0.821**	**0.851**	**0.451**	**0.850**	**7.0**	**2.33**

Bold values highlight the proposed YOLO-RSTS model, emphasizing its effectiveness and facilitating intuitive comparison.

The results in the table demonstrate that YOLO-RSTS consistently surpasses the compared lightweight YOLO variants on key segmentation metrics while requiring substantially less computation. Specifically, compared with YOLOv8n, YOLO-RSTS increases recall from 0.710 to 0.821 and mAP0.50 from 0.810 to 0.851, with GFLOPs reduced from 12.0 to 7.0. Compared with YOLOv10n, YOLO-RSTS increases precision by approximately 5.4%, recall by approximately 9.3%, and mAP0.50 by approximately 7.6%, while reducing GFLOPs by about 17.6% and parameter count by about 15.3%. Compared with YOLO11n, precision improves by roughly 11.1%, recall by roughly 4.6%, and mAP0.50 by roughly 5.7%, with GFLOPs reduced by approximately 31.4% and parameters reduced by approximately 18.0%. Notably, relative to the most recent baseline YOLOv13n, YOLO-RSTS yields about a 7.4% increase in precision, a 16.8% increase in recall, a 9.7% increase in mAP0.50 and a 10.3% increase in mAP0.50:0.95, while decreasing GFLOPs by approximately 30.7% and parameter count by approximately 13.7%. Overall, these percentage improvements indicate that YOLO-RSTS achieves consistent and meaningful performance gains across lightweight YOLO variants.

From a low-level architectural perspective, YOLO-RSTS is built on a YOLOv12n backbone and integrates CrossScaleDSC and C2f-DSC, CG-Attention, and lightweight primitives RFCAConv and DWConv to enhance multi-scale detail representation and contextual awareness under strict lightweight constraints. Compared with other YOLO variants, YOLOv8 (C2f/SPPF/FPN-/PAN-style necks) improves cross-stage reuse and multi-scale feature extraction but remains dominated by local convolutions and therefore provides limited long-range context; YOLOv10 (C2f/RepNCSP-ELAN/PSA/SCDown) aggressively pursues compactness (e.g., SCDown’s spatial–channel decoupled downsampling and NMS-free assignment) to minimize FLOPs, yet such simplification can weaken inter-layer interaction and local representation, degrading performance in complex backgrounds and along fine boundaries; YOLO11 (C3k2/SPFF/C2PSA/Transformer backbone with dynamic heads) strengthens fine-grained representation through richer fusion and parallel attention at the cost of increased module complexity and compute, which complicates full global coverage and lightweight deployment; YOLOv12 emphasizes attention-centric non-local modeling to improve global consistency but substantially increases memory, computation, and implementation complexity; YOLOv13 (HyperACE/FullPAD/DS-based blocks/Gated Fusion) captures high-order cross-scale relations via hypergraph modeling and full-pipeline aggregation while using DS blocks to reduce cost, yielding robustness in complex scenes but with reduced cost-effectiveness in low-compute or small-data settings. To address these limitations, YOLO-RSTS adopts a coordinated modular strategy. CrossScaleDSC and C2f-DSC preserve and strengthen cross-stage, multi-scale information flow while substantially reducing parameters and computation through depth-separable convolutions, thereby providing more direct and engineering-friendly cross-layer interaction than v8’s standard C2f re-parameterization/reversible schemes at comparable FLOPs. CG-Attention injects long-range semantics via a compact channel–space coupling that approximates the global benefits of v12/v13 at a fraction of the cost, avoiding the memory and latency penalties of full global correlation or hypergraph computations. Concurrently, RFCAConv and DWConv reduce the cost of local filtering and downsampling, preserving or improving local representations and freeing budget for cross-stage aggregation and attention. As a result, unlike v10’s extreme compression or YOLO11/v12’s heavy attention, YOLO-RSTS maintains robust cross-layer information and context awareness while remaining efficient and deployment-friendly. By balancing cross-stage consistency, long-range context compensation, and local aggregation cost control, YOLO-RSTS achieves an optimal lightweight trade-off: in resource-constrained engineering scenarios it attains context gains comparable to heavy-attention designs with fewer parameters and lower compute, thereby offering a practical performance advantage over other YOLO-series variants.

### 4.4 Recall-Precision curve comparison on the dataset

**Figure 6:**
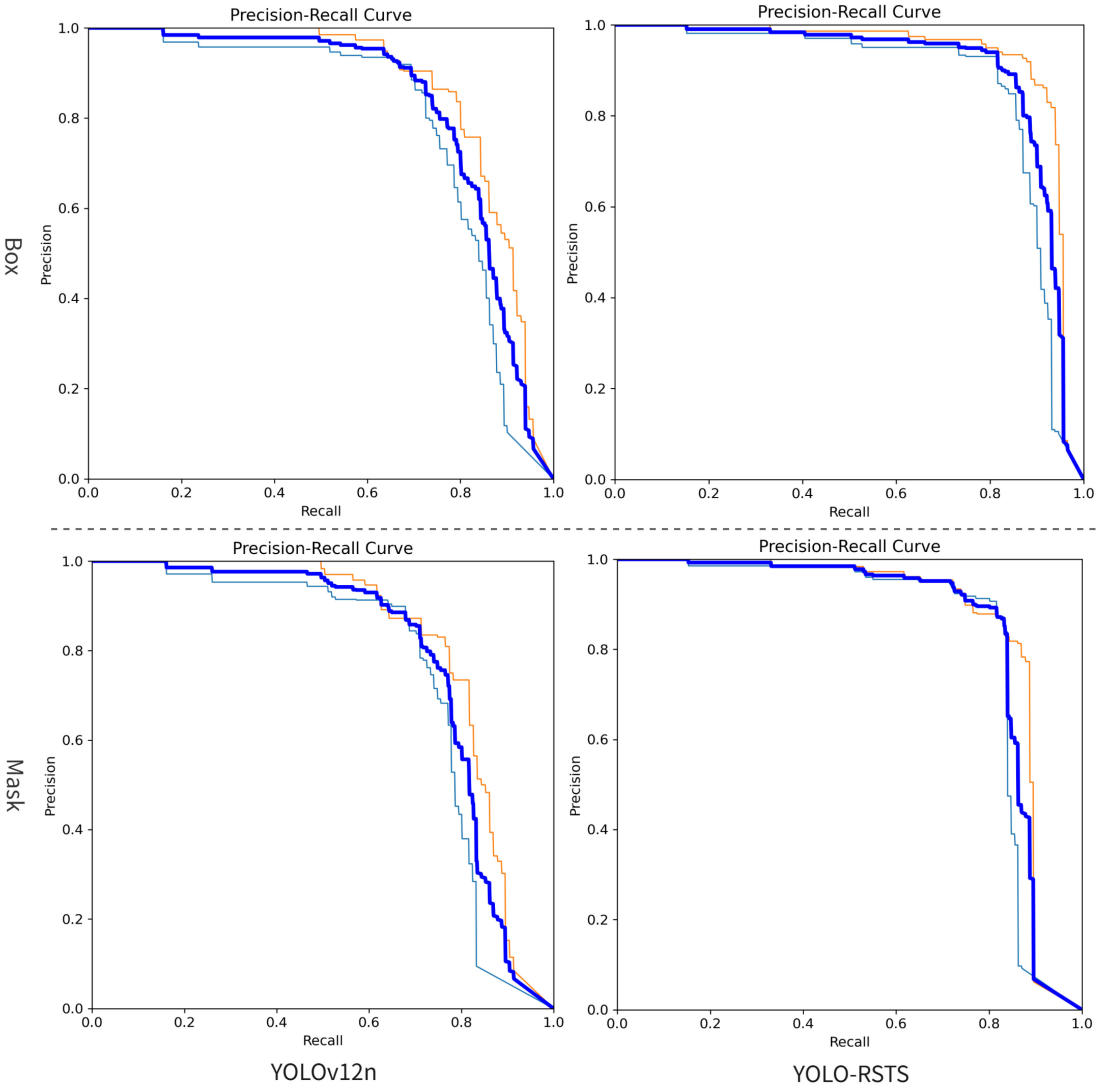
The comparison of the Recall-Precision curves.

To better illustrate the performance differences between YOLO-RSTS and the baseline YOLOv12n, we plot their corresponding Precision–Recall (P–R) curves based on the experimental results from the dataset, as shown in **Figure 6**. This visualization helps reveal how each model balances precision and recall across various confidence thresholds, providing a more intuitive and detailed understanding of their detection behavior.

In the figure, the right column shows the proposed YOLO-RSTS and the left column shows the baseline YOLOv12n; the top row presents Precision–Recall curves for bounding-box detection, while the bottom row shows Precision–Recall curves for mask segmentation. YOLO-RSTS’s curves lie consistently closer to the upper-right corner, yielding higher precision at equivalent recall, an advantage that is particularly pronounced in the high-recall regime (recall is greater than 0.8), which indicates reduced missed detections while maintaining low false-positive rates. The curves are also more compact and exhibit lower variance, implying reduced sensitivity to the confidence threshold and greater operational stability, thereby facilitating selection of robust operating points and reducing tuning overhead. Improvements in the mask curves further demonstrate enhanced pixel-level segmentation quality and boundary consistency. Overall, YOLO-RSTS attains a substantially larger area under the Precision–Recall curves than the baseline, corresponding to higher AP and mAP and superior overall performance.

### 4.5 Demonstration of segmentation performance

**Figure 7:**
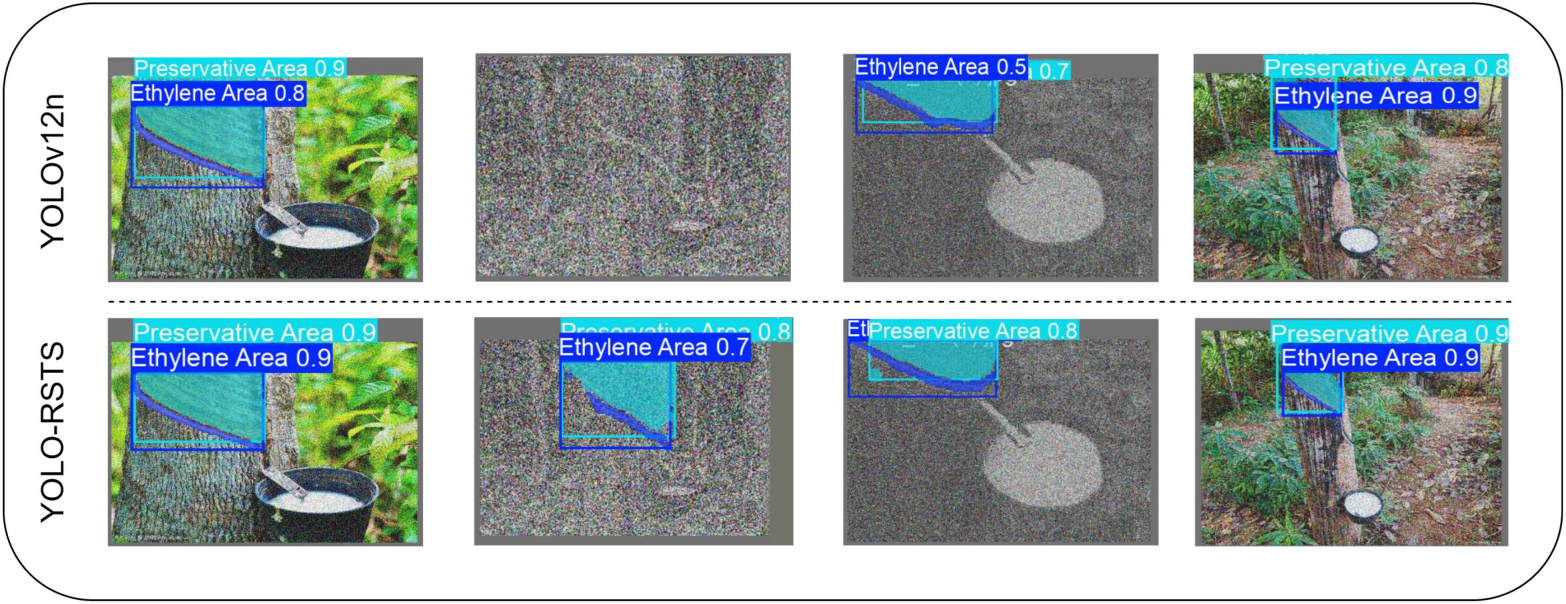
Demonstration of segmentation performance comparison between YOLO-RSTS and YOLOv12n model.

For a more intuitive comparison of segmentation performance between the proposed YOLO-RSTS and the original YOLOv12n, this study randomly sampled a set of rubber-tree images from the test set. The results are shown in **Figure 7**.

From the comparison illustrated in the figure, it is evident that YOLO-RSTS demonstrates several significant advantages over the original YOLOv12n in terms of segmentation results. Firstly, YOLO-RSTS exhibits a more precise fitting of object boundaries, with its segmentation masks aligning more closely with manually annotated contours, thereby significantly reducing missed detections at the edges. Secondly, it shows improved connectivity recovery for small-scale targets and fine branches, effectively mitigating common issues of fragmentation and disconnection observed in YOLOv12n while maintaining overall mask integrity. Furthermore, in complex scenarios characterized by similar background textures or occluded objects, YOLO-RSTS displays enhanced robustness, leading to a noticeable reduction in both false positives and missed detections. Visually, the masks generated by this model exhibit less noise and smoother contours; additionally, the overlay visualization results facilitate subsequent precision automation spraying tasks. Overall, the excellent segmentation accuracy and robustness of YOLO-RSTS demonstrate its potential for applications in automated pesticide spraying scenarios, enabling more precise application of rubber pesticides and effective reduction of chemical usage.

### 4.6 Generalization evaluation on Hainan field dataset

To further evaluate the robustness and cross-regional generalization capability of the YOLO-RSTS model, we conducted a comprehensive validation using an independently collected test dataset from major natural rubber plantation areas in Hainan Province. Compared with the training domain, this region exhibits substantial differences in climate conditions, illumination patterns, plant morphology, growth stages, and background clutter, thereby providing an effective basis for assessing detection stability under out-of-distribution (OOD) scenarios. The cross-regional evaluation incorporates multiple performance metrics, including overall accuracy and coverage, class-specific accuracy and detection coverage for different regions (Preservative and Ethylene), as well as efficiency indicators such as total inference time, per-image processing latency, and throughput. In addition, detection-stability metrics—including the number of images with valid detections, the number of images without any detections, and the total number of high-confidence detections—are used to further assess the model’s practical applicability.

**Figure 8:**
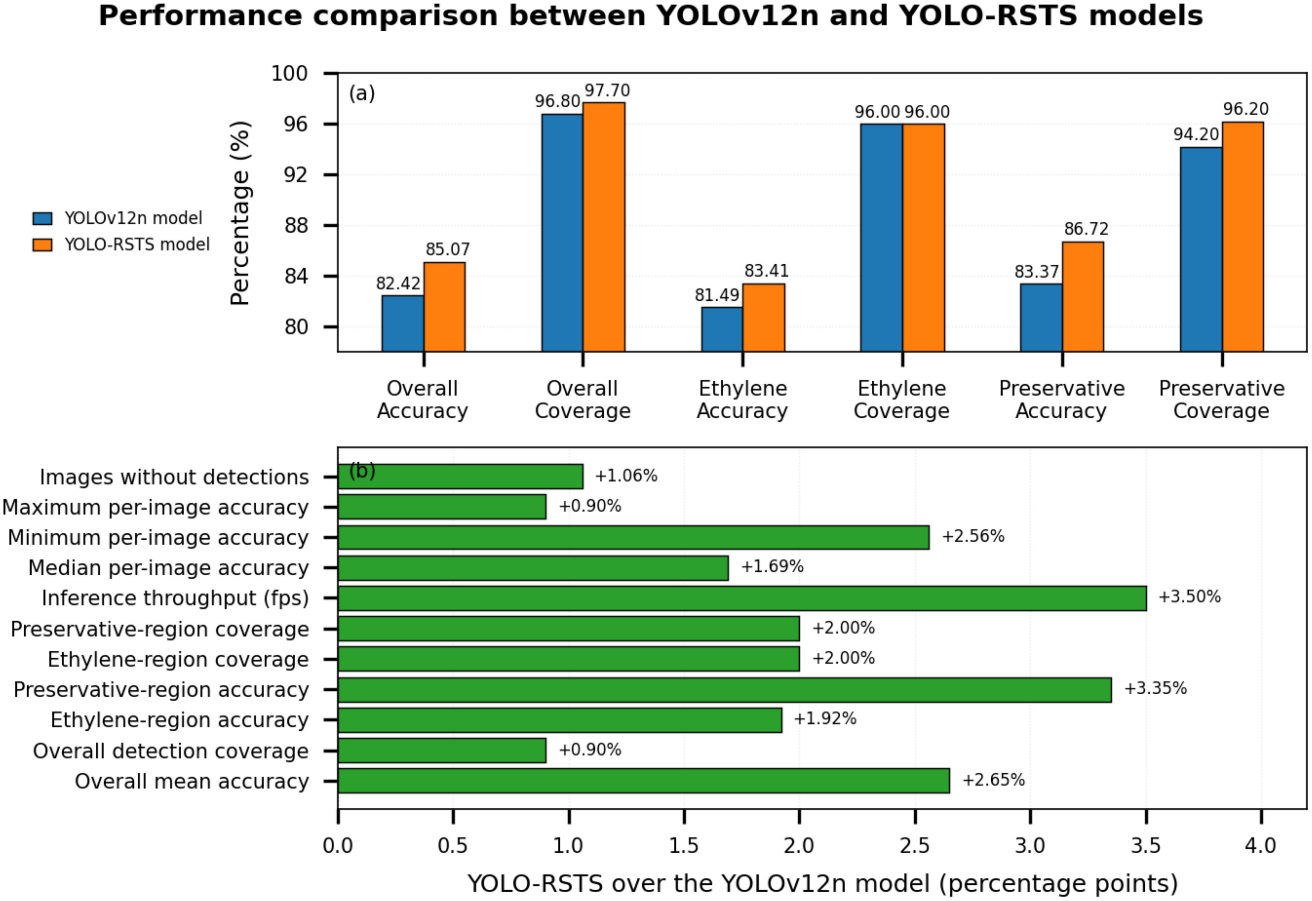
Intuitive visual comparison of the cross-regional performance between the YOLOv12n model and the improved YOLO-RSTS model on the Hainan independent test set (valid detections only). **(a)** presents an image-level comparison of accuracy and coverage between YOLOv12n and YOLO-RSTS for ethylene and preservative regions. **(b)** illustrates the relative performance gains of YOLO-RSTS over YOLOv12n across key image-level and detection-related metrics.

To preclude biased conclusions and ensure more rigorous characterization, this study conducted experiments and statistically analyzed results under two complementary evaluation schemes. First, an image-level analysis (**Table 4** and **Figure 8**) computes, for each image and each category, the best detection accuracy and then derives summary statistics such as overall average accuracy, median accuracy, regional coverage, and class-wise performance. This protocol focuses on valid detections and effectively excludes false detections when aggregating the best-case per-image performance, making it suitable for assessing whether the model can reliably capture the target regions at least once per image. Second, a detection-level analysis (**Table 5**) aggregates statistics over all predicted boxes, explicitly including false detections and repeated detections, and evaluates average, median, minimum, and maximum detection-level accuracy, as well as class-wise mean accuracy and changes in the number of images with or without detections. This protocol is more sensitive to error patterns and confidence distribution, and thus reflects the model’s behavior under dense, potentially noisy prediction scenarios. Together, image-level best detection metrics and detection-level full detection statistics complement each other, providing a more comprehensive and statistically grounded evaluation of the improved YOLO-RSTS model’s cross-regional reliability, robustness, and deployment potential in practical production environments.

**Table 4:** Accuracy-distribution, inference-efficiency, and detection-stability metrics for the YOLOv12n and improved YOLO-RSTS models on the Hainan independent test set (valid detections only).

Metric	YOLOv12n model	YOLO-RSTS model	Improvement (%)
Accuracy distribution statistics			
Median accuracy	0.8460	0.8687	+2.68
Minimum accuracy	0.4002	0.4171	+4.22
Maximum accuracy	0.9385	0.9641	+2.73
Standard deviation	0.0856	0.0880	+2.80
Inference efficiency			
Total inference time (s)	28.86	27.86	+3.47 (faster)
Time per image (s/image)	0.083	0.081	+2.41 (faster)
Inference throughput (fps)	11.99	12.41	+3.50 (faster)
Detection statistics			
Images with valid detections	335	338	+0.90
Images without detections	11	8	−27.27
Total high-confidence detections	658	665	+1.06

**Table 5:** Accuracy-distribution, inference-efficiency, and detection-stability metrics for the YOLOv12n and improved YOLO-RSTS models on the Hainan independent test set (including false detections).

Metric	YOLOv12n model	Improved YOLO-RSTS	Improvement (%)
Overall detection statistics			
Images with detections	338	340	+0.59
Images without detections	8	6	−25.00
Time per image (s)	0.081	0.079	+2.47 (faster)
Inference throughput (fps)	12.41	12.64	+1.85 (faster)
Overall detection-level accuracy			
Average accuracy	0.7876	0.8142	+3.38
Median accuracy	0.8369	0.8620	+3.00
Minimum accuracy	0.2517	0.2521	+0.16
Maximum accuracy	0.9385	0.9641	+2.73
Per-class accuracy statistics			
Preservative area accuracy	0.8041	0.8334	+3.64
Ethylene area accuracy	0.7720	0.7951	+2.99

The results in **Table 4** summarize the image-level performance, where for each image and each region (ethylene and preservative), only the best valid detection is considered. This protocol evaluates whether the model can correctly identify each target region at least once per image, without being influenced by repeated detections or false positives. Under this evaluation, the improved YOLO-RSTS model shows consistent gains: median accuracy increases from 0.8460 to 0.8687, maximum accuracy rises from 0.9385 to 0.9641, and the number of images with valid detections increases from 335 to 338. Inference speed also improves, with per-image latency reduced from 0.083,s to 0.081,s. These results indicate that the refined model exhibits stronger reliability at the level of overall image interpretation.

**Table 5** also presents experimental results encompassing all predicted bounding boxes, regardless of whether they represent duplicate detections, missed detections, or false positives. This protocol reflects the raw output distribution of the detector and is more sensitive to prediction noise. Even under this stricter evaluation, the YOLO-RSTS model still shows clear advantages: average detection-level accuracy increases from 0.7876 to 0.8142 (+3.38%), median accuracy improves by +3.00%, and images without any detections decrease from 8 to 6 (–25%). Class-wise performance also improves, with preservative-area accuracy rising by +3.64% and ethylene-area accuracy by +2.99%. At the same time, per-image inference time improves from 0.081,s to 0.079,s., confirming that accuracy improvements do not come at the expense of computational efficiency.

Although the improved YOLO-RSTS model achieves measurable reductions in per-image inference latency, the overall acceleration appears modest when assessed at the batch level. This is primarily due to the batch-wise testing protocol adopted in this study, in which all 346 images were processed within a single execution pipeline. Under this setting, a substantial portion of the total runtime is dominated by fixed computational and system-level overheads such as GPU context initialization, memory allocation, kernel warm-up, data loading, and I/O synchronization. These operations impose nearly identical time costs on both models, which weakens the observable difference in the actual forward-pass computation. As a result, despite the fact that YOLO-RSTS provides a measurable per-image speed improvement, the acceleration becomes less evident when assessed at the batch scale.

Even with these inherent limitations, the bar charts (a) and (b) in the figure show that the refined YOLO-RSTS model still delivers consistent and meaningful efficiency gains, reflected in its slight reduction in per-image latency and a 3.50% increase in throughput. More importantly, the improvements extend well beyond computational efficiency. Median, minimum, and maximum per-image accuracies all increase, indicating a more stable and reliable prediction distribution with fewer low-quality outputs. Detection stability also improves, with fewer missed detections and higher numbers of valid, high-confidence predictions. Furthermore, the model achieves substantial gains in critical regional metrics, particularly the 3.35% improvement in preservative-region accuracy, which highlights its enhanced ability to distinguish fine-grained features under diverse conditions.

Taken together, these results confirm that the enhanced YOLO-RSTS model achieves not only higher accuracy and improved detection stability but also retains its computational advantages even when evaluated under batch-processing constraints. The consistent performance gains observed on the independent Hainan test set, which features climatic, phenotypic, and environmental characteristics distinct from the training domain, provide strong evidence that the model maintains robust cross-regional generalization capability. These findings suggest that the refined YOLO-RSTS model remains well suited for deployment in real-world rubber plantation monitoring systems operating across diverse geographical regions.

In addition to the aforementioned statistical evaluation, to more rigorously test the model’s performance in unfamiliar scenarios, we specifically selected 100 images from the Hainan test set that exhibit significant visual differences from the training set (Xishuangbanna). These images were meticulously manually annotated to construct a high-quality supervised test subset. Based on this, the study also conducted a supplementary inference validation. Unlike the previous evaluation, which relied on model inference on unlabeled field images and image-level statistics, the following inference validation is grounded in precise manual annotations. Consequently, this inference experiment can provide a stricter and more fine-grained measurement of segmentation quality, yielding more convincing results.

As shown in **Table 6**, the YOLO-RSTS model achieves strong accuracy on this manually annotated subset, with a Box mAP0.50 of 0.873 and a precision of 0.847. Several factors help explain these results. First, inference was performed on an NVIDIA RTX 3090 (24 GB) with a batch size of 16, which differs from the hardware and parameter settings used during training and earlier evaluations. Such hardware-and batch-related variations often lead to slight numerical improvements. Second, the manually annotated subset contains clearer and more representative spraying regions, which reduces ambiguity compared with the broader unlabeled field-scale evaluation. Consequently, the resulting mAP0.50 is broadly consistent with the overall performance of YOLO-RSTS on the Xishuangbanna dataset (0.851) and falls within the expected range. Moreover, it is close to the median accuracy of 0.8687 reported in **Table 4**, further confirming the validity and reliability of the earlier accuracy statistics obtained without newly annotated images, and strengthening confidence in the overall performance of the YOLO-RSTS model.

**Table 6:** Supervised evaluation of YOLO-RSTS on the newly annotated Hainan subset.

Class	Instances	Precision	Recall	F1 score	mAP0.50	mAP0.50:95
all	200	0.847	0.877	0.862	0.873	0.564
Ethylene Area	100	0.892	0.913	0.902	0.943	0.654
Preservative Area	100	0.801	0.840	0.820	0.804	0.473

Overall, both the statistical evaluation based on unlabeled images and the supervised evaluation based on manually labeled samples demonstrate the effectiveness of the application of YOLO-RSTS in Hainan rubber growing areas. The consistency between these two validation approaches confirms that the model maintains stable accuracy across different geographic and seasonal conditions, reinforcing its strong cross-regional generalization capability and practical suitability for deployment in real rubber plantation environments.

## 5 Discussion

This study adopts an improved YOLO-RSTS segmentation model to perform intelligent recognition and segmentation of the preservative and stimulant spraying areas on rubber trees. By introducing three innovative modules—CrossScaleDSC, CG-Attention, and C2f-DSC—the model significantly improves the precision of identifying and distinguishing spraying areas, even in the challenging rubber plantation environment. Experimental results show that, compared to existing methods, the proposed model achieves significant improvements in mean precision (increased from 0.788 to 0.851) and recall (increased from 0.740 to 0.821), while reducing the model size by 14.5%. The improvements in segmentation precision and efficiency provide valuable contributions to optimizing pesticide spraying operations in rubber plantations, ultimately supporting more efficient and sustainable plantation management.

Despite the initial progress made in pesticide spraying in rubber plantations, several real-world challenges remain, especially in terms of background interference and area segmentation accuracy. Firstly, compared to other crops, the environment of rubber plantations is more complex, with dense canopies, thick and irregularly shaped trunks, and varying latex flow areas, making the structure and spraying zones of each tree highly diverse. These factors increase the difficulty of target recognition and area segmentation, particularly in high-density plantations where the distinction between the target objects and the background is often minimal, leading to a reduced accuracy of traditional methods (Lei et al., 2024). Notably, in the areas where stimulant is sprayed, the narrowness of the zones and their similarity to the surrounding background often make them difficult to distinguish effectively. This issue is similar to the fine-grained target problem in weed segmentation, where small and irregularly shaped targets cause a substantial drop in segmentation accuracy (Luo et al., 2025). Furthermore, relying solely on visible-light images for precise segmentation presents a performance limit in complex environments. Rubber plantations are typically exposed to open environments where variations in lighting, shadow interference, and reflection effects significantly affect image quality. As the light changes throughout the day, shadows cast by tree trunks and leaves often blur the target areas, interfering with area recognition and lowering segmentation accuracy. Additionally, under harsh weather conditions, such as rain or haze, the visibility of visible-light images may worsen, leading to loss or misjudgment of visual information, which weakens the model’s robustness in real-world applications (Arsalan et al., 2024). Therefore, current recognition solutions need further optimization to develop more robust and adaptable intelligent spraying area recognition models and solutions that can handle the complexities of environmental changes.

In addition to the practical challenges discussed above, the model design and comparison strategy in this study were also constrained by the computational realities of rubber plantation applications. In automated on-board spraying systems, inference must be performed on resource-limited edge devices under strict latency and power constraints, which fundamentally limits the feasible complexity of the segmentation framework. According to the official reports of mainstream segmentation models, many advanced instance and panoptic segmentation frameworks—such as Mask R-CNN (He and Gkioxari, 2018), HTC (Chen et al., 2019), SOLOv2 (Wang et al., 2020), and Mask2Former (Cheng et al., 2022)—achieve strong performance on general vision benchmarks, yet their parameter scales and FLOPs are typically one to two orders of magnitude larger than those of lightweight YOLO-based architectures. For example, conventional Mask R-CNN and HTC models often exceed 40–60M parameters and require more than 150–250 GFLOPs per inference, whereas our YOLO-RSTS model contains only approximately 2.33M parameters and requires merely 7.0 GFLOPs, making it substantially more suitable for real-time deployment on edge devices used in rubber plantation spraying systems. Additional pilot experiments on the Hainan rubber plantation test set (not reported here due to the limited interpretability of comparing with such heavyweight models) further indicated that these large frameworks provide only marginal gains in segmentation accuracy while requiring several-fold increases in inference time and memory consumption. As a result, direct comparisons with YOLO-RSTS—which is explicitly designed for real-time field deployment—would be neither meaningful nor fair. Given these constraints, we therefore focused our baseline comparisons on the YOLO segmentation family, whose architectures share similar design principles regarding real-time performance and computational efficiency. This choice allows performance differences to be interpreted primarily as algorithmic improvements rather than artifacts of vastly different computational budgets, and it better reflects the practical requirements of intelligent on-board spraying in rubber plantations. These findings also suggest that future research should further explore models that can not only improve segmentation accuracy but also further maintain deployability under the complex and resource-constrained conditions of real-world plantation environments.

Beyond the previously discussed design constraints, the improved YOLO-RSTS model also demonstrates strong generalization when evaluated in the rubber-growing regions of Hainan, even though the testing conditions differ markedly from those of the Xishuangbanna training environment in both geography and season. This stability can be attributed to the inherent similarities shared among major tropical rubber-producing regions. Both Xishuangbanna and Hainan exhibit comparable climatic conditions, characterized by high temperatures, high humidity, and year-round evergreen vegetation, which result in similar bark textures, latex flow patterns, and overall visual features of rubber trees. The tapping techniques practiced in both regions also follow standardized industry procedures, producing consistent incision orientations, flow-mark structures, and stimulant application regions. Plantation layout and tree spacing are likewise largely uniform, further contributing to the similarity of trunk-level spraying regions that dominate the visual input of the segmentation task. Although the two regions differ in topography, with Xishuangbanna being predominantly mountainous and Hainan relatively flat, these landscape variations exert minimal influence on model performance because image acquisition focuses primarily on the trunk surface rather than the broader environment. Therefore, these ecological and operational consistencies across Yunnan’s Xishuangbanna region and Hainan Province offer a coherent explanation for the robust cross-regional and cross-seasonal performance of YOLO-RSTS and support its suitability for deployment across diverse rubber plantation environments.

Future research will focus on developing target segmentation models that are better adapted to the unique environmental conditions of rubber plantations, with an emphasis on integrating recent state-of-the-art technologies. These innovations can effectively address the complex ecological challenges of rubber plantations and advance agricultural production toward greater precision and intelligence. In the domain of deep learning, emerging models based on self-attention mechanisms, such as Vision Transformer (ViT) and Swin Transformer, will enable more flexible global context modeling and local feature fusion, thereby enhancing segmentation accuracy, particularly under conditions involving overlapping traces and highly variable illumination (Zeng et al., 2024) (Liu et al., 2023) (Chen et al., 2023),,. When combined with architectures such as U-MixFormer and CSWin-UNet, these approaches can effectively handle intricate environmental details and fine-scale harvesting regions while simultaneously improving computational efficiency and model scalability (Cao et al., 2022). From a data fusion perspective, the integration of multispectral imaging with LiDAR technology is expected to further boost segmentation accuracy, as multispectral imaging facilitates the identification of subtle texture variations on rubber tree surfaces, whereas LiDAR provides precise three-dimensional spatial information for accurate modeling of target regions (Zhang et al., 2024) (Wang et al., 2023),. Furthermore, super-resolution reconstruction techniques and deep learning–based small-object detection algorithms will strengthen the recognition of narrow and fine spraying areas, enabling even more refined agricultural operations. On the hardware side, the deep integration of edge computing and the Internet of Things (IoT) will drive the full automation of large-scale spraying operations, allowing real-time monitoring of environmental changes and dynamic adjustment of spraying strategies (Panda et al., 2023). Ultimately, the convergence of these advanced technologies will not only improve spraying efficacy and minimize pesticide waste but also enable more precise and environmentally friendly agricultural management, fostering the continued development of intelligent and precision-oriented rubber plantation management.

## 6 Conclusion

This paper proposes YOLO-RSTS, a high-precision segmentation model for identifying stimulant and preservative spraying areas on rubber trees. Built on a YOLOv12n-Seg backbone, the model integrates our proposed CrossScaleDSC, CG-Attention, and C2f-DSC modules, adopts RFCAConv to expand the receptive field and introduce channel attention, and replaces standard convolutions with DWConv to reduce parameters and computational cost. These complementary components work synergistically to produce more coherent and accurate segmentation under complex bark textures, elongated spray bands, and strong lighting or background interference, while maintaining a lightweight design.

A comprehensive suite of ablation and model comparison experiments was conducted to evaluate YOLO-RSTS and its constituent modules. Relative to YOLOv12n, the proposed model improves Precision by 6.3%, Recall by 8.1%, and mAP0.50 by 6.3%, while reducing parameters by 14.5%; compared with the newer YOLOv13n, it achieves gains of 6.1% in Precision, 11.8% in Recall, and 4.2% in mAP0.50, together with a 3.7% reduction in parameters. These results, further supported by precision–recall curves and qualitative visualizations, demonstrate clear advantages in detection quality, boundary stability, and computational efficiency. In addition to in-distribution evaluations, the model was further tested on a newly collected cross-regional and cross-seasonal dataset from Hainan, which differs markedly from the Xishuangbanna training environment in climate, season, phenology, and background complexity. The consistent improvements observed on this OOD dataset confirm that YOLO-RSTS maintains strong generalization capability across geographic and seasonal domains.

Taken together, these findings demonstrate that YOLO-RSTS delivers the accuracy, robustness, and real-time performance required for deployment in real rubber plantation environments. Its reliable detection capabilities across diverse conditions provide a strong foundation for automated spraying systems, enabling improved operational efficiency, reduced labor dependence, and more precise, sustainable plantation management. These strengths position YOLO-RSTS as a practical and promising solution for advancing intelligent rubber plantation operations.

## Data Availability

The raw data supporting the conclusions of this article will be made available by the authors, without undue reservation.
