# Supplementary material for: YOLO-RSTS: a precise segmentation model for detecting preservative and stimulant spraying regions on rubber trees
Source: Front Plant Sci. 2026 Jan 7;16:1738496. doi: 10.3389/fpls.2025.1738496 (PMC12819190; doi:10.3389/fpls.2025.1738496)
Supplement: Supplementary file 1 [file DataSheet1.pdf]

Supplementary Material for YOLO-RSTS: A Precise Segmentation Model for Detecting Preservative and Stimulant Spraying Regions on Rubber Trees

1 INTRODUCTION

2 METHODS

2.1 Innovative CrossScaleDSC module

2.2 Innovative CPCA-GAM module

2.3 Innovative C2f-DSC module

2.4 Introduced RFCAConv module

To enhance the accurate segmentation of spraying regions on rubber trees, particularly in distinguishing between preservative and stimulant areas, the RFCAConv module (1) was integrated into the detection model. This module utilizes a multi-scale receptive field to effectively capture fine details such as spray boundaries and bark irregularities, as well as broader contextual information such as variations in tree texture and spray coverage. Furthermore, its channel attention mechanism selectively enhances critical features, such as the subtle distinction between sprayed and non-sprayed regions, ensuring robust performance under complex and dynamic environmental conditions, including changing lighting and intricate backgrounds. The internal structure of the RFCAConv is shown in the Figure S1.

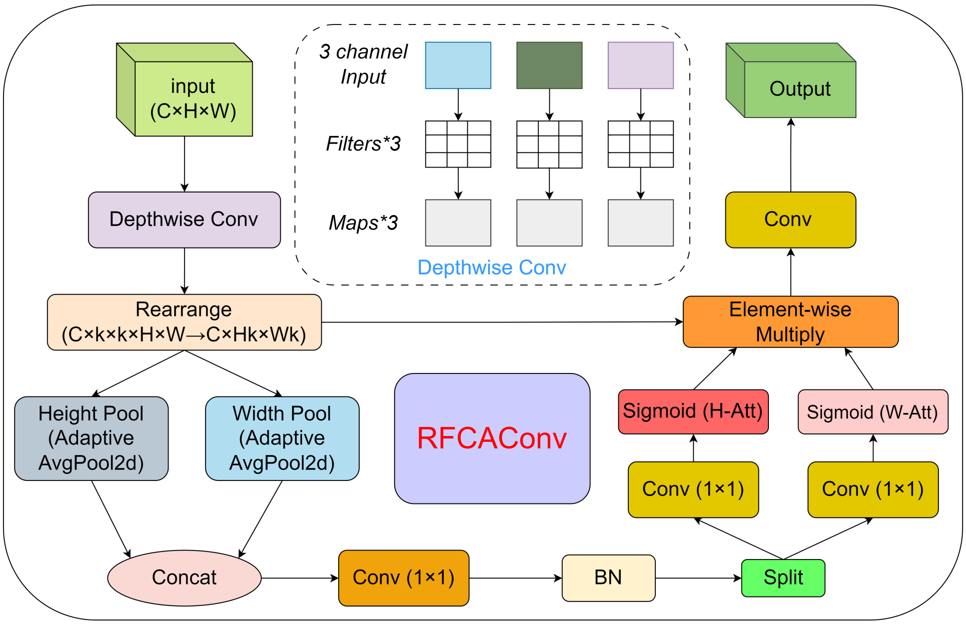

Figure S1. Internal structure diagram of RFCAConv module.

The RFCAConv module begins by applying depthwise convolutions to the input feature map. The input is a tensor $X \in \mathbb{R}^{C \times H \times W}$, where C denotes the number of channels, and H and W represent the spatial dimensions. Each channel c is convolved with its own unique kernel $K^{(c)}$, resulting in the following depthwise convolution operation:

$$X_{\text{dw}}^{(c)} = K^{(c)} * X^{(c)}, \quad \text{for } c = 1, 2, \dots, C$$

This depthwise convolution preserves the number of channels while enhancing spatial features.

Next, RFCAConv performs adaptive average pooling separately along the height and width dimensions to capture directional dependencies across the spatial layout. These pooling operations generate directional feature representations as follows:

$$F_h = \text{AvgPool}_H(X_{\text{dw}}) \in \mathbb{R}^{C \times H_k \times 1}, \quad F_w = \text{AvgPool}_W(X_{\text{dw}}) \in \mathbb{R}^{C \times 1 \times W_k}$$

These pooled feature tensors are then rearranged and concatenated to form a unified channel-wise descriptor F_{cat} for attention modeling:

$$F_{\text{cat}} = \text{Concat}(\text{Rearrange}(F_h), \text{Rearrange}(F_w)) \in \mathbb{R}^{C \times (H_k + W_k)}$$

The concatenated tensor F_{cat} undergoes a 1×1 convolution followed by batch normalization (BN) to refine the feature map. This refined map is split into two branches to generate directional attention maps for the height and width dimensions:

$$F = \text{BN}(\text{Conv}_{1\times1}(F_{\text{cat}}))$$

$$A_h = \sigma(\text{Conv}_{1\times1}(F_h)), \quad A_w = \sigma(\text{Conv}_{1\times1}(F_w))$$

Here, $\sigma(\cdot)$ denotes the sigmoid activation function, and A_h and A_w are the attention maps for the height and width dimensions, respectively.

These attention maps are then expanded back to the original feature dimensions and applied to the depthwise convolution output via element-wise multiplication:

$$X_{\text{rfca}} = X_{\text{dw}} \odot A_h \odot A_w$$

Finally, a convolution layer is applied to the attention-weighted features to generate the module's output:

$$\text{Output} = \text{Conv}(X_{\text{rfca}})$$

This approach integrates multi-scale receptive fields with directional attention, enabling RFCAConv to significantly improve the segmentation accuracy, especially in distinguishing the preservative-treated and stimulant-treated regions on the rubber tree bark.

2.5 Introduced Depth-wise Conv

To optimize computational efficiency while preserving accuracy, the Depthwise Convolution (DWConv)(2) module integrates two primary operations: depthwise convolution (3) and pointwise convolution (4). The structure diagram of DWConv is shown in the Figure S2.

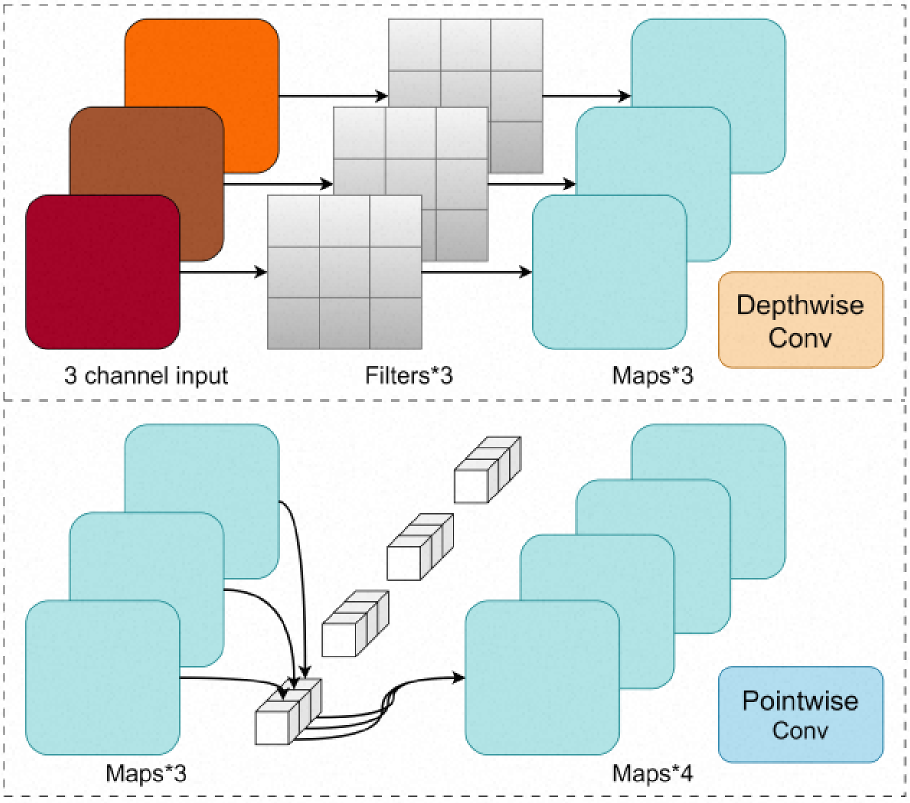

Figure S2. Internal structure diagram of DWConv module.

In the depthwise convolution stage, each input channel is processed independently with a unique filter (e.g., $K \times K$ kernels such as 3×3). This operation focuses on extracting spatial features within each individual channel, avoiding any interaction between the channels themselves. For instance, if the input has dimensions of $H \times W \times 3$, three separate filters are applied (one for each channel), resulting in an output with dimensions of $H' \times W' \times 3$. This decoupling of channels significantly reduces the number of parameters and floating-point operations (FLOPs) compared to conventional convolutions, where cross-channel interactions are involved.

Afterward, in the pointwise convolution stage, a set of 1×1 filters is used to combine the depthwise outputs across the different channels. This operation allows for the fusion of features across channels while enabling flexibility in adjusting the output's depth (i.e., the number of channels). For example, applying four $1 \times 1 \times 3$ filters to the depthwise output of size $H' \times W' \times 3$ results in a feature map of size $H' \times W' \times 4$. This approach maintains the original spatial resolution while expanding the feature representation into a higher-dimensional space, enriching the model's capacity to learn more complex patterns.

In this study, the DWConv structure is particularly well-suited for tasks such as precise segmentation of spray boundaries in rubber trees, where both high computational efficiency and accurate feature extraction

are crucial. DWConv is capable of efficiently processing complex spatial information while preserving the ability to capture fine-grained details, making it highly suitable for real-time image analysis in agricultural applications such as rubber tree spraying area segmentation.

3 DATASETS AND EVALUATION INDICATORS

4 EXPERIMENTS AND RESULTS

4.1 Configuration of Experimental Environment

4.2 Ablation Experiments

4.2.1 Ablation Experiments on the Dataset

4.2.2 Comparison of C2f-CPCA with Other Feature Fusion Modules

4.3 Model Comparison Experiments

4.4 Recall-Precision Curve Comparison on the Dataset

4.5 Comparison of mAP0.50 and mAP0.50:0.95 Performance Trends Across YOLO Models Over Epochs

In order to gain a deeper understanding of the performance of the improved YOLO-RSTS and various YOLO models throughout the training process, this study presents a comparative visualization of the YOLOv10n, YOLOv11n, YOLOv12n, and the improved YOLO-RSTS models, utilizing two key performance metrics: mAP0.50 and mAP0.50-0.95. The mAP0.50 metric reflects the average precision of the model when detecting objects at a 0.50 threshold, while mAP0.50-0.95 provides a more comprehensive evaluation, showcasing the model's performance across multiple thresholds ranging from 0.50 to 0.95. By comparing these metrics, we can more precisely analyze and evaluate how these models improve their detection performance as the number of epochs increases during training. The visualization result is shown in the Figure S3.

From the Figure S3, it is evident that the improved YOLO-RSTS model significantly outperforms the other three YOLO versions in both mAP0.50 and mAP0.50-0.95, especially in the later stages of training, where the performance gains of YOLO-RSTS are most pronounced. While in the early training phases, the performance of YOLO-RSTS is comparable to that of YOLOv12n, as training progresses, YOLO-RSTS consistently increases its mAP scores, ultimately surpassing the other models. This indicates that YOLO-RSTS has clear advantages in model generalization and detection accuracy, allowing it to maintain high efficiency across a broader range of detection tasks. Notably, the gradual improvement in the mAP0.50-0.95 metric further demonstrates the stability and robustness of YOLO-RSTS at varying confidence thresholds, validating its ability to adapt to object detection in complex environments.

These visualization results further emphasize the effectiveness of the architectural improvements in YOLO-RSTS, reinforcing its status as an advanced model for real-world rubber spraying region segmentation tasks.

4.6 Demonstration of Segmentation Performance

4.7 Generalization Evaluation on Hainan Field Dataset

5 DISCUSSION

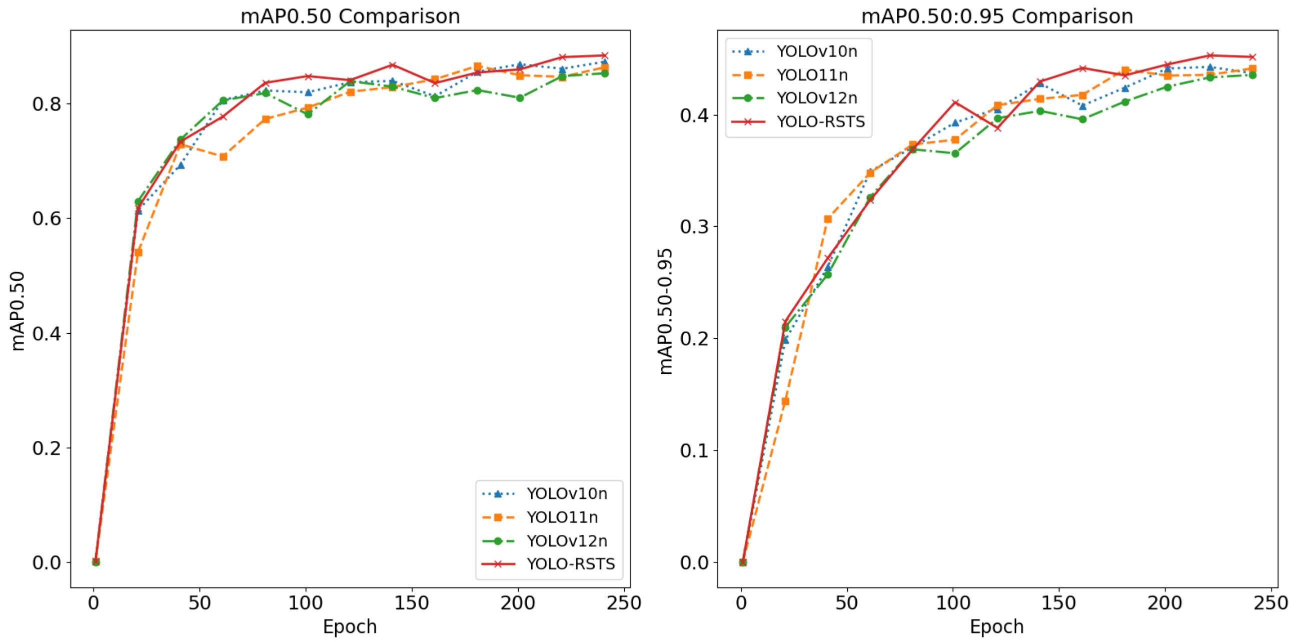

Figure S3. Evolution of mAP0.50 and mAP0.50:0.95 for YOLO models over training epochs.

6 CONCLUSION

REFERENCES

[1]X. Zhang, C. Liu, D. Yang, T. Song, Y. Ye, K. Li, and Y. Song, "RFCAConv: Innovating spatial attention and standard convolutional operation," *arXiv preprint arXiv:2304.03198*, 2023.

[2]F. Chollet, "Xception: Deep learning with depthwise separable convolutions," in *Proceedings of the IEEE Conference on Computer Vision and Pattern Recognition*, 2017, pp. 1251–1258.

[3]Y. Guo, Y. Li, L. Wang, and T. Rosing, "Depthwise convolution is all you need for learning multiple visual domains," in *Proceedings of the AAAI Conference on Artificial Intelligence*, vol. 33, no. 01, 2019, pp. 8368–8375.

[4]B.-S. Hua, M.-K. Tran, and S.-K. Yeung, "Pointwise convolutional neural networks," in *Proceedings of the IEEE Conference on Computer Vision and Pattern Recognition*, 2018, pp. 984–993.
